# Cezanne is a critical regulator of pathological arterial remodelling by targeting β-catenin signalling

**DOI:** 10.1093/cvr/cvab056

**Published:** 2021-02-18

**Authors:** Weiwei An, Le A Luong, Neil P Bowden, Mei Yang, Wei Wu, Xinmiao Zhou, Chenxin Liu, Kaiyuan Niu, Jun Luo, Cheng Zhang, Xiaolei Sun, Robin Poston, Li Zhang, Paul C Evans, Qingzhong Xiao

**Affiliations:** 1 Centre for Clinical Pharmacology, William Harvey Research Institute, Barts and The London School of Medicine and Dentistry, Queen Mary University of London, Charterhouse Square, London EC1M 6BQ, UK; 2 Department of Infection, Immunity and Cardiovascular Disease, Bateson Centre, and Insigneo Institute for In Silico Medicine, University of Sheffield, Beech Hill Rd, Sheffield S10 2RX, UK; 3 Department of Cardiology, and Institute for Cardiovascular Development and Regenerative Medicine, Xinhua Hospital Affiliated to Shanghai Jiaotong University School of Medicine, Shanghai, China; 4 Centre for Microvascular Research, William Harvey Research Institute, Barts and The London School of Medicine and Dentistry, Queen Mary University of London, London, UK; 5 Key Laboratory of Cardiovascular Diseases at The Second Affiliated Hospital, School of Basic Medical Sciences, Guangzhou Medical University, Guangzhou, Guangdong, China; 6 Guangzhou Municipal and Guangdong Provincial Key Laboratory of Protein Modification and Degradation, School of Basic Medical Sciences, Guangzhou Medical University, Guangzhou, Guangdong, China

**Keywords:** Cezanne, Vascular smooth muscle cells, Neointima, Post-angioplasty restenosis, Atherosclerosis, Cell proliferation, Cell migration

## Abstract

**Aims:**

Pathological arterial remodelling including neointimal hyperplasia and atherosclerosis is the main underlying cause for occluding arterial diseases. Cezanne is a novel deubiquitinating enzyme, functioning as a NF-кB negative regulator, and plays a key role in renal inflammatory response and kidney injury induced by ischaemia. Here we attempted to examine its pathological role in vascular smooth muscle cell (VSMC) pathology and arterial remodelling.

**Methods and results:**

Cezanne expression levels were consistently induced by various atherogenic stimuli in VSMCs, and in remodelled arteries upon injury. Functionally, VSMCs over-expressing wild-type Cezanne, but not the mutated catalytically-inactive Cezanne (C209S), had an increased proliferative ability and mobility, while the opposite was observed in VSMCs with Cezanne knockdown. Surprisingly, we observed no significant effects of Cezanne on VSMC apoptosis, NF-κB signalling, or inflammation. RNA-sequencing and biochemical studies showed that Cezanne drives VSMC proliferation by regulating CCN family member 1 (CCN1) by targeting β-catenin for deubiquitination. Importantly, local correction of Cezanne expression in the injured arteries greatly decreased VSMC proliferation, and prevented arterial inward remodelling. Interestingly, global Cezanne gene deletion in mice led to smaller atherosclerotic plaques, but with a lower level of plaque stability. Translating, we observed a similar role for Cezanne in human VSMCs, and higher expression levels of Cezanne in human atherosclerotic lesions.

**Conclusion:**

Cezanne is a key regulator of VSMC proliferation and migration in pathological arterial remodelling. Our findings have important implications for therapeutic targeting Cezanne signalling and VSMC pathology in vascular diseases.

## Graphical Abstract

**Figure:**
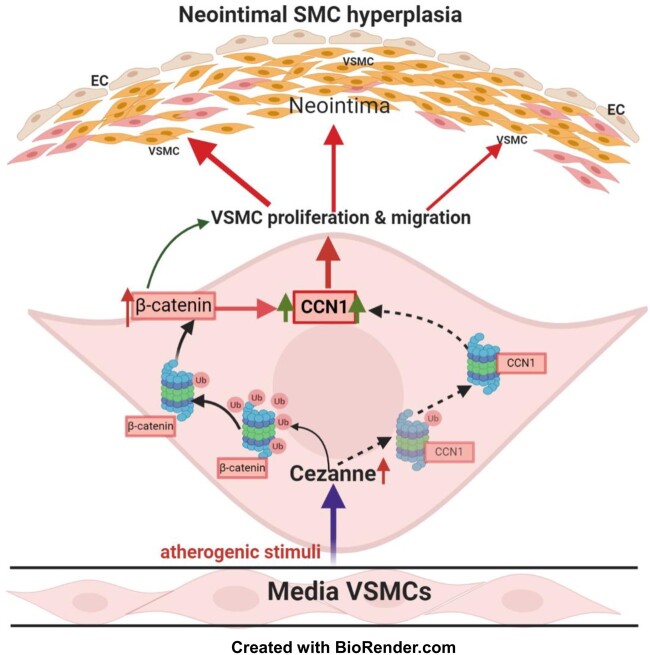

**Time for primary review: 44 days**

## 1. Introduction

Cardiovascular disease (CVD) is one of the leading causes for death worldwide, and pathological arterial remodelling including neointimal hyperplasia and atherosclerosis is a key determinant of these life-threatening conditions. Therefore, understanding and controlling pathological arterial remodelling is urgently required to manage patients with CVDs. Vascular smooth muscle cell (VSMC) is the major cells in the media layer of all the blood vessels, with critical roles in vasodilation and vasoconstriction. In response to injury and inflammation, VSMCs become activated and switch to a synthetic phenotype and contribute to vascular remodelling or neointimal SMC hyperplasia.^1^ As such, dysfunctional VSMCs have been implicated in many vascular diseases including atherosclerosis,^2–5^ and fine-tuning regulation of VSMC function represents a therapeutic possibility for post-angioplasty restenosis and other CVDs.

Protein ubiquitination, and its reversal by protein de-ubiquitination via deubiquitylating enzymes (DUBs), plays a critical role in governing all essential cellular functions and controlling a variety of biological processes.^6^^,^^7^ One of the DUBs, cellular zinc finger anti-NF-κB (Cezanne) has been first cloned and characterized as a negative regulator of the NF-κB signalling pathway by us in 2001.^8^ We were the first to demonstrate that Cezanne is a novel DUB, which can cleave ubiquitin monomers from ubiquitinated proteins.^9^ Subsequently, we have shown that Cezanne is induced by multiple proinflammatory cytokines and, as an inhibitor of NF-κB signal pathway, it forms a negative feedback loop in inflammatory cytokine signalling.^10^^,^^11^ Moreover, Cezanne can be induced by hypoxia in endothelial cells through p38MAPK–dependent transcriptional and post-transcriptional mechanisms, and by ischaemia in the murine kidney, and Cezanne regulates inflammatory responses to hypoxia by targeting tumour necrosis factor receptor–associated factor (TRAF) 6 for de-ubiquitination.^12^ Interestingly, a recent study from another group revealed that Cezanne controls noncanonical NF-κB activation through deubiquitination of TRAF3.^13^ Pathophysiologically, Cezanne has been reported to regulate cancer progression^14^ and cell survival,^15^ and to be involved in the neuronal hyperplasia through modulating the Notch signalling pathway.^16^ However, there is no report about the functional involvements of Cezanne in VSMC pathology and arterial remodelling in the literature. Here, we have provided clear evidence that Cezanne is a key regulator of VSMC proliferation/migration and arterial remodelling, and it regulates CCN1 expression through controlling Wnt/β-catenin signalling pathway by targeting β-catenin for deubiquitination.

## 2. Methods

### 2.1 Animal experiments, anaesthesia, and euthanasia

All animal experiments were conducted according to the Animals (Scientific Procedures) Act of 1986 (United Kingdom). All the animal procedures were approved by Queen Mary University of London ethics review board (PPL number: PB508B78D), and conform to the guidelines from Directive 2010/63/EU of the European Parliament on the protection of animals used for scientific purposes or the NIH guidelines (Guide for the care and use of laboratory animals). For mouse femoral artery denudation injury and local gene transfer, anaesthesia was induced using 100% O_2_/4% isoflurane, and was maintained throughout the procedure by the administration of 100% O_2_/2% isoflurane. At the end of protocol, all mice were euthanized by placing them under deep anaesthesia with 100% O_2_/5% isoflurane, followed by decapitation.

### 2.2 Mouse femoral artery denudation injury and shRNA lentiviral particle infusion

Eight-week-old male C57BL/6 mice were anaesthetized and the surgical procedure was similar to that described previously.^17–19^ Briefly, the femoral artery was dissected and injured by passing the tip (0.23 mm) of angioplasty spring-wire (Hi-Torque Winn 200 T guide wire, Stock Number: 1012474, Abbott Laboratories. Illinois, USA) for three to five times. After endothelium denudation, the injured arteries were randomly incubated with sh-NT or sh-Cez lentivirus. The procedures for local SMC infection were similar to that described in our previous studies^20^^,^^21^ with some modifications. In brief, immediately after injury, 10–20 µL of DMEM containing 1.0–2.0 × 10^6^ sh-NT or sh-Cez lentiviral particles was directly infused into the lumen of the injured femoral arteries, followed by a 30-min incubation for local VSMC infection. As we previously reported with GFP lentivirus,^21^ local infusion of the lentivirus in the injured arteries mainly results in SMC infection, with no or minimum gene transduction in other aortic cells.

### 2.3 Analysis of human femoral and coronary arteries

Human normal healthy and diseased femoral arteries were collected and described in our previous study,^17^ and human coronary arterial biopsy specimens^22^^,^^23^ were collected by Dr Robin Poston. Briefly, human arterial specimens were obtained from patients with peripheral arterial diseases undergoing leg amputation at the First Affiliated Hospital of Zhejiang University (China) between July 2014 and June 2017. All patients gave their written, informed consent prior to inclusion in the study. All studies were approved by the Research Ethics Committees of the First Affiliated Hospital of Zhejiang University (2013/150), and all experiments were conducted according to the principles expressed in the Declaration of Helsinki. It is worth noting that the human coronary arterial autopsy specimens were harvested with permission by Dr Robin Poston, a cardiovascular pathologist, in the Department of Pathology, Guy’s Hospital, London (1980–1990). The currently required study approval for collecting such specimens was not applicable since the regulatory Human Tissue Act was not enacted until 2004 in the UK. Standard immunofluorescence analysis was conducted on paraffin sections of human femoral and coronary arteries to examine respective protein expression as described in Supplementary material online.

### 2.4 Statistical analysis

Results are presented as mean ± standard error of the mean (SEM). Statistical analysis was performed using Graphpad Prism-8.3. Shapiro–Wilk Normality Test was used for checking the normality of the data, and all the data have passed the test. Two-tailed unpaired student’s *t*-test was used for comparisons between two groups, or one-/two-way analysis of variance with a post hoc test of Tukey’s analysis was applied when more than two groups were compared. *P* < 0.05 was considered statistically significant.

### 2.5 Additional materials and methods

Additional detailed description of materials and methods is provided in Supplementary material online.

## 3. Results

### 3.1 Cezanne expression in VSMCs

**Figure 1:**
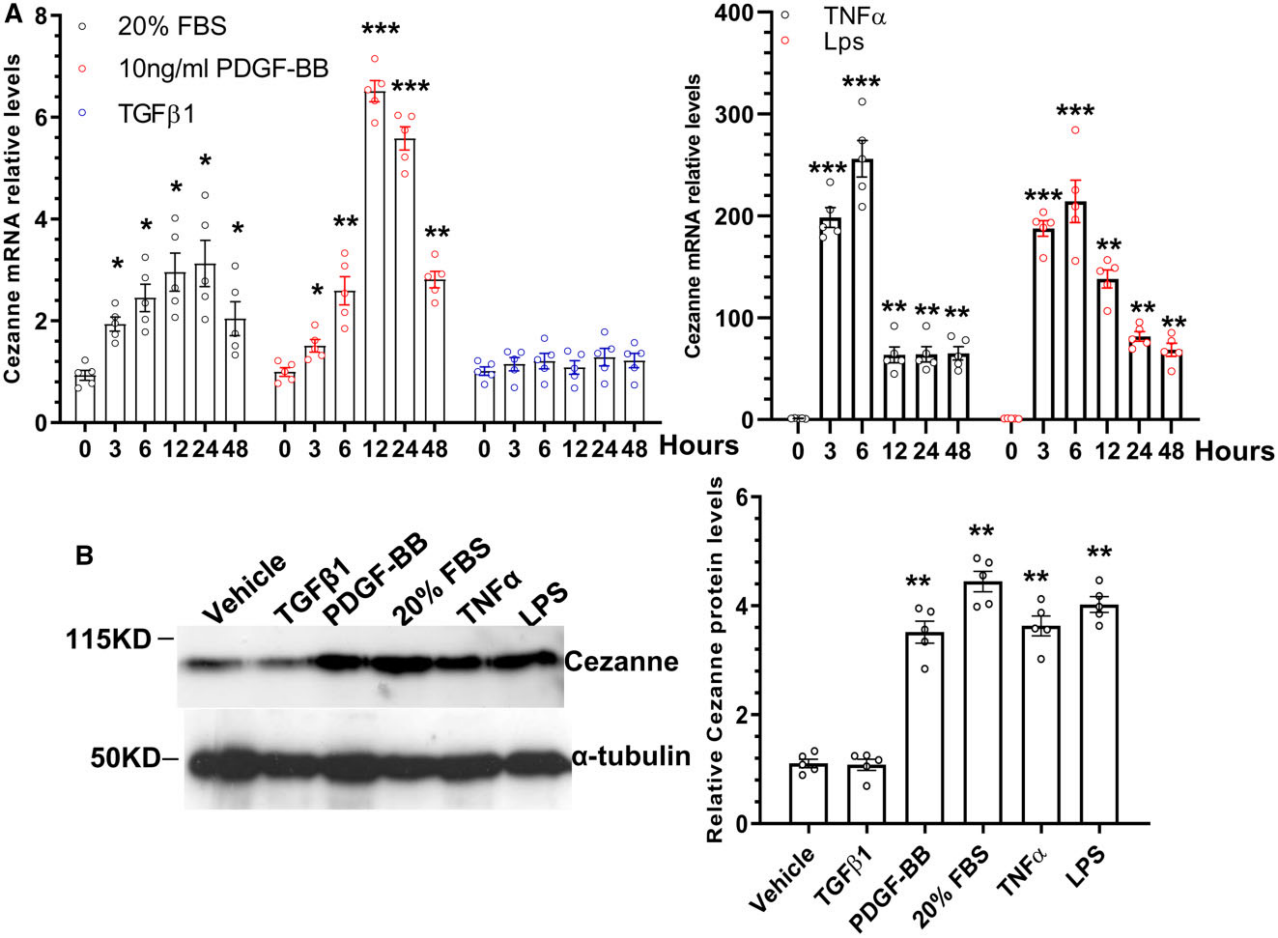
Cezanne expression is up-regulated in VSMCs treated with various atherogenic stimuli. Cultured VSMCs (p5–p8) were subjected to serum starvation for 48 h, followed by stimulation with 20% FBS, 10 ng/mL PDGF-BB, 5 ng/mL TGFβ1, 50 ng/mL TNFα, or 1000 ng/mL Lps for the indicated times. Total RNAs and protein (12 h) were harvested and subjected to RT-qPCR (*A*) and western blotting (*B*) analyses, respectively. The data presented here are representative (left in *B*) or mean ± SEM of five independent experiments (*n* = 5). *P < 0.05, **<0.01, ***<0.001 (vs. 0 h or vehicle) (one-way ANOVA with a *post hoc* test of Tukey’s analysis).

As reported previously, Cezanne can be induced by hypoxia in endothelial cells.^12^ However, little is known about its expression and modulation in VSMCs. We therefore first investigated if Cezanne expression could be modulated by various atherogenic stimuli. Our data showed that Cezanne was significantly up-regulated in VSMCs in response to 20% foetal bovine serum (FBS), 10 ng/mL platelet-derived growth factor BB (PDGF-BB), 50 ng/mL TNFα, and 1000 ng/mL lipopolysaccharides (LPS) treatments, while its expression level was not changed in response to 5 ng/mL transforming growth factor beta-1 (TGFβ1) (*Figure 1*) stimulation. As expected, we observed highest Cezanne expression in response to both TNFα and LPS stimulation, which peaked at 6 h and reduced thereafter. These data suggest that Cezanne plays a role in VSMC responses to pathological factors.

### 3.2 Cezanne controls VSMC proliferation and migration

**Figure 2:**
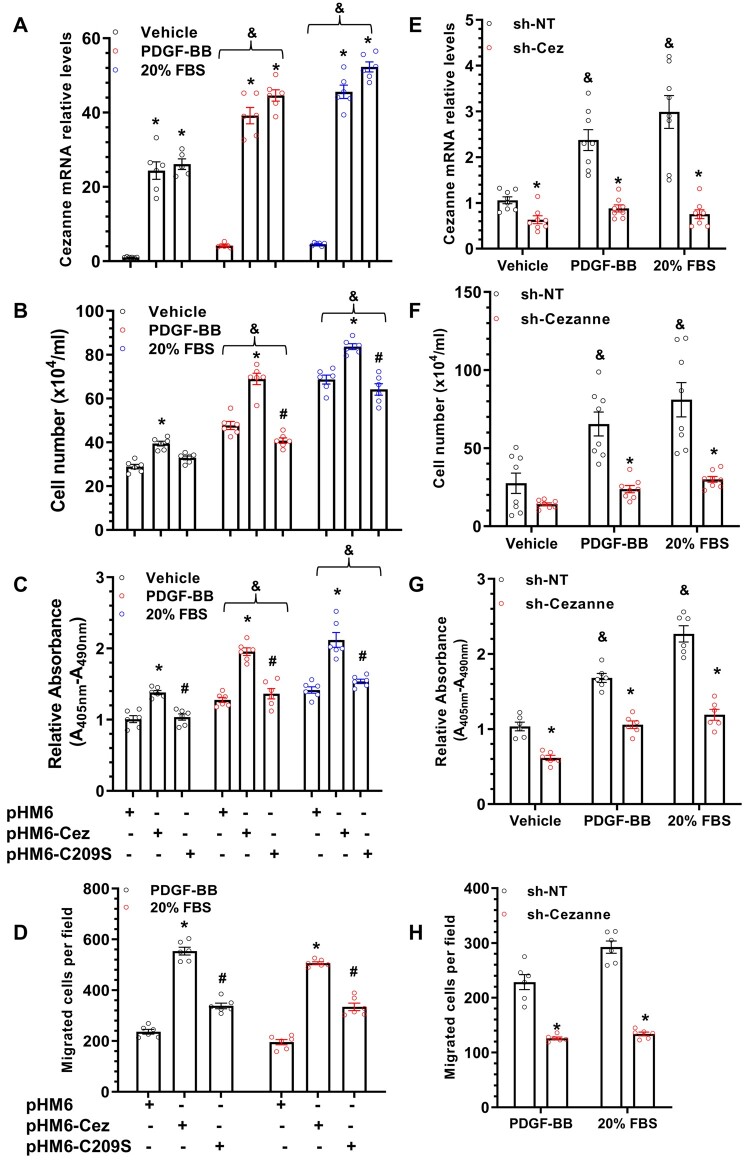
Cezanne regulates VSMC proliferation and migration. (*A–D*) Cezanne over-expression regulated VSMC functions. VSMCs transfected with control (pHM6), wild-type (pHM6-Cez), or mutated (pHM6-C209S) Cezanne plasmids were subjected to serum starvation for 48 h, followed by 10 ng/mL PDGF-BB, or 20% FBS stimulations for another 12 (migration) or 24 (proliferation) hours, respectively. (*A*) RT-qPCR analysis of Cezanne gene expression. (*B and C*) Cezanne over-expression promoted VSMC proliferation as demonstrated in total cell number count (*B*) and BrdU incorporation assay (*C*). (*D*) Cezanne increases VSMC migration as demonstrated in transwell migration assays. Note: no or only a few cells were migrated through the inserts without a strong chemoattractant in the bottom chamber; therefore, no control treatment is shown in transwell migration assays. (*E–H*) Cezanne gene knockdown in VSMCs. VSMCs infected with non-target (sh-NT) or Cezanne specific (sh-Cez) shRNA lentivirus were subjected to a similar treatment and analysis as described in (*A–D*). The data presented here are mean ± SEM of six (*n* = 6 in *A–D* and *G–H*) or eight (*n* = 8 in E–F) independent experiments. *P < 0.05 (vs. pHM6 or sh-NT); ^#^*P* < 0.05 (pHM6-C209S vs. pHM6-Cez); ^&^*P* < 0.05 (vs. vehicle) (two-way ANOVA with a *post hoc* test of Tukey’s analysis).

To explore a role of Cezanne in various VSMC functions, VSMCs were transfected with control (pHM6), wild-type (pHM6-Cez), or mutated Cezanne (pHM6-Cez-C209S, Cys209 is a catalytic residue of Cezanne, which is responsible for the deubiquitinating activity of Cezanne against both linear and branched forms of polyubiquitin^9^) plasmid, and subjected to various treatments and analyses as indicated. As expected, the expression of Cezanne was successfully up-regulated in VSMCs in the absence or presence of various stimulations (*Figure 2A* and Supplementary material online, *Figure S1A***)**. Importantly, Cezanne over-expression in VSMCs significantly increased their proliferation under base level as well as in response to both PDGF-BB and FBS stimulations, while such an increase was almost abolished by over-expression of the mutated Cezanne (pHM6-Cez-C209S) (*Figure 2B and C*). Similar effects were observed in VSMC migration upon both PDGF-BB and FBS stimulations (*Figure 2D* and Supplementary material online, *Figure S1B*). These data indicate that Cezanne plays an important role in VSMC growth and mobility, and its deubiquitinating activity is responsible for such effects. To further confirm the role of Cezanne in VSMC proliferation and migration, loss-of-function experiments were conducted using Cezanne shRNA in VSMCs, followed by similar treatments and assays. As shown in *Figure 2E* and Supplementary material online, *Figure S1E*, the endogenous levels of Cezanne in VSMCs were significantly down-regulated by its shRNAs. Consequently, VSMC proliferation (*Figure 2F–G*) and migration (*Figure 2H* and Supplementary material online, *Figure S1F*) were significantly inhibited once endogenous Cezanne was inhibited, further confirming a role for Cezanne in VSMC proliferation and migration.

### 3.3 Cezanne plays an insignificant role in VSMC apoptosis, VSMC-specific gene expression, NF-kB signalling, and inflammatory response in VSMCs

Apart from proliferation and migration, VSMC apoptosis also play a role in determining vascular pathological response after injury. We therefore wondered if Cezanne could play a role in VSMC apoptosis. To this end, similar Cezanne gain/loss-of-function experiments were conducted as previously described. TUNEL staining assays showed that VSMC apoptosis induced by combinational treatment with 100 ng/mL TNFα and 200 ng/mL interferon gamma (IFN-γ) was not impaired by either Cezanne overexpression (Supplementary material online, *Figure S1C and D*) or inhibition (Supplementary material online, *Figure S1G and H*). Similarly, VSMC phenotype modulation is a well-known mechanism underlying arterial remodelling upon injury. We thus examined if SMC-specific gene expression were controlled by Cezanne. For this purpose, VSMCs were transfected with Cezanne over-expression plasmids, and subjected to TGFβ1 treatment to induce SMC differentiation. Gene expression data (Supplementary material online, *Figure S2*) showed that Cezanne was successfully up-regulated by both wild type and mutated Cezanne plasmids in VSMCs. As expected, a variety of SMC marker gene expressions (SMαA, SM22α, h1-calponin, SMTN-B, and SM-myh11) were significantly induced by TGFβ1, while such gene inductions were not impaired by Cezanne over-expression (Supplementary material online, *Figure S2*), suggesting that Cezanne plays no role in TGFβ1-induced SMC gene expressions in VSMCs. Finally, VSMC inflammatory response is another major determinant for neointimal SMC hyperplasia in response to vascular injury, and functioning as NF-кB negative regulator Cezanne has been recognized as one of major anti-inflammatory regulators. We therefore speculated that Cezanne may play a role in VSMC inflammatory response. Surprisingly, although the NF-кB reporter activity was hugely increased by both TNFα and Lps in VSMCs, their activity was not impaired by Cezanne over-expression (Supplementary material online, *Figure S3A*), suggesting that Cezanne plays no role in NF-кB signalling activation in VSMCs. Such a notion was further supported by gene expression data (Supplementary material online, *Figure S3B–H*). Specifically, while the gene expression levels of all the inflammatory genes (MCP-1, iNOS, ICAM-1, VCAM-1, E-selectin, and IL6) examined in this study were significantly up-regulated by both inflammatory stimuli, no apparent effect of Cezanne over-expression on these gene expression was observed (Supplementary material online, *Figure S3C–H*). Taken together, above data have collectively demonstrated that Cezanne plays no significant role in VSMC death, TGFβ1-induced SMC gene expressions, and inflammatory response.

### 3.4 RNA sequencing analysis to uncover the potential downstream targets of cezanne and associated signal pathways in VSMCs

In searching the potential downstream target genes of Cezanne in VSMCs, total RNAs were extracted from VSMCs infected with control and Cezanne knockdown shRNA lentivirus and subjected to RNA sequencing analyses. A total of 75 genes were significantly modulated by Cezanne knockdown in VSMCs, with 18 up-regulated and 57 down-regulated as shown in the Heatmap (Supplementary material online, *Figure S4A*). Panther GO-Slim analysis showed that these modulated genes could be categorized into seven GO-Slim molecular functions including catalytic activity, binding, and transporter activity (Supplementary material online, *Figure S4B*), fifteen GO-Slim biological processes (e.g. cellular process, biological regulation, metabolic process, locomotion, and growth) (Supplementary material online, *Figure S4C*), and 10 protein classes (e.g. metabolite interconversion enzyme, intercellular signal molecule, protein modifying enzyme, extracellular matrix protein, cytoskeletal protein, and nucleic acid binding protein) (Supplementary material online, *Figure S4D*), respectively. The top enriched GO terms in the three main domains of molecular function, biological process, and protein class were shown in Supplementary material online, *Figure S4E*. Among them, five GO terms were in the domain of molecular function, which were mostly related to heparin/integrin binding, and cell adhesion molecule binding; six GO terms were in the domain of biological process, which were mainly related to regulation of cell migration, supramolecular fibre organization, and cell death/adhesion; seven GO terms were related to protein class, which were associated with growth factor, extracellular matrix structural protein, extracellular matrix protein, and general transcription factor.

### 3.5 Ccn1 is the top downstream target gene modulated by Cezanne in VSMCs

**Figure 3:**
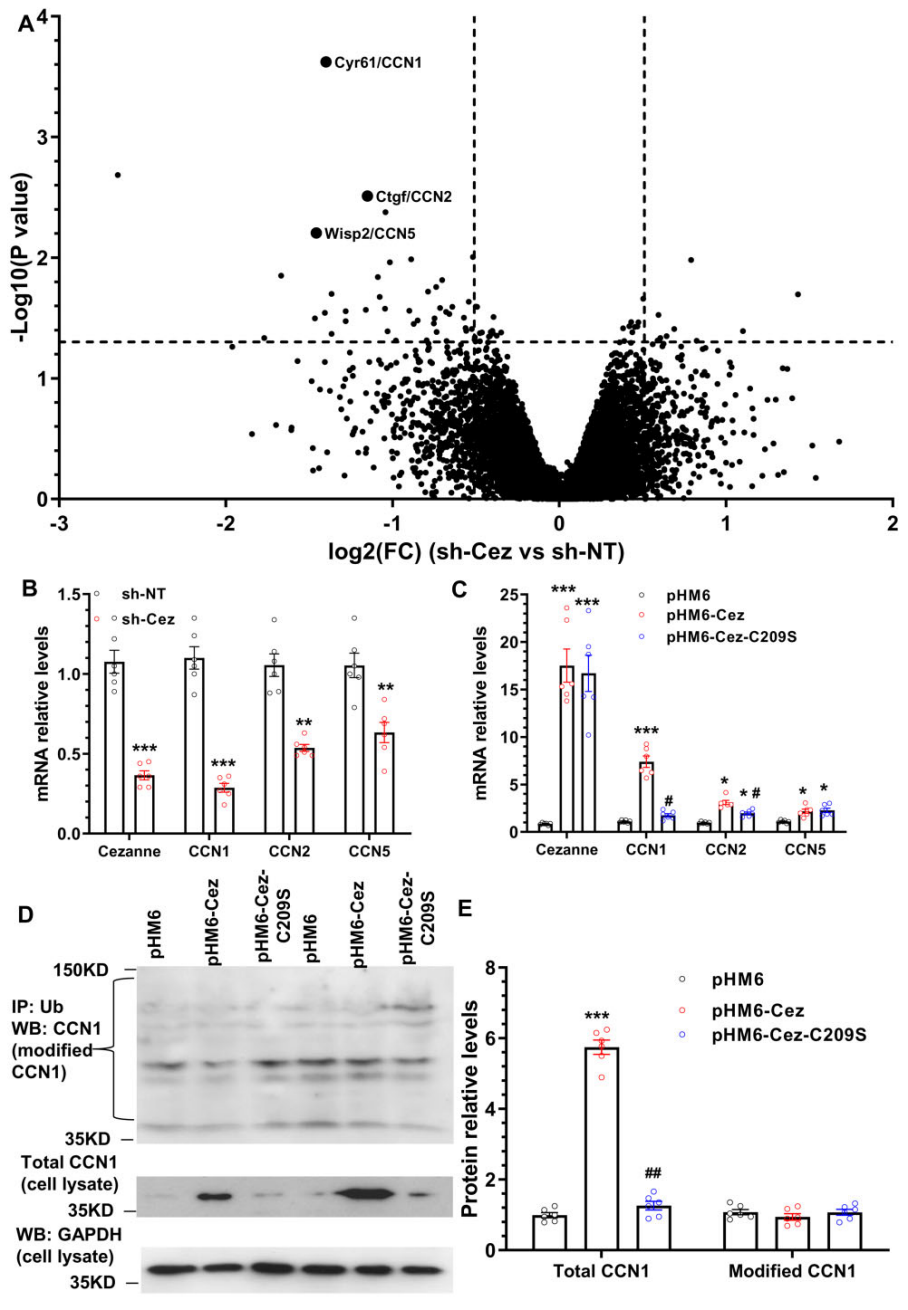
Cezanne regulates CCN1 but not targets it for deubiquitination in VSMCs. (*A*) Volcano plot analysis showing three CCN genes modulated by Cezanne knockdown in VSMCs. Total RNAs were isolated from VSMCs infected with non-target (sh-NT) or Cezanne specific (sh-Cez) shRNA lentivirus and subjected to RNA-sequencing analysis, followed by Volcano plot analysis. (*B*) RT-qPCR analysis validated CCN genes expression. (*C*) CCN genes expression was regulated by Cezanne. (*D–E***)** The expression level of total, but not the ubiquitinated CCN1 protein was regulated by Cezanne. VSMCs transfected with control (pHM6), wild-type (pHM6-Cez) or mutated (pHM6-Cez-C209S) Cezanne plasmids were subjected to serum starvation for 48 h, followed by 50 ng/mL TNFα stimulations for another 6 h. After then, cells were treated with 20 µM MG132 for 1 h. Pan-ubiquitin immunoprecipitates or total cell lysates were tested by western blotting using anti-CCN1 antibody to examine ubiquitinated (modified CCN1) and total CCN1 protein, respectively. The data presented here are representative (left panel in *D*) or mean ± S.E.M. of three (*A, n* = 3) or six (*B–E, n* = 6) independent experiments. *P < 0.05, **<0.01, ***<0.001 (vs. sh-NT or pHM6); ^#^*P* < 0.05, ^##^<0.01 (pHM6-Cez-C209S vs. pHM6-Cez) (*B*, unpaired *t*-test; *C* and *E*, one-way ANOVA with a *post hoc* test of Tukey’s analysis).

Interestingly, as shown in the volcano plot analysis three CCN family members including CCN1, CCN2 and CCN5 were down-regulated by Cezanne inhibition (*Figure 3A*), which was further confirmed in RT-qPCR analysis (*Figure 3B*). Importantly, Cezanne over-expression in VSMCs significantly increased all three CCN genes expression, while such up-regulations for CCN1 and CCN2, not for CCN5 gene was almost abolished by over-expression of the mutated Cezanne (*Figure 3C*). In particular, CCN1 or cysteine-rich angiogenic inducer 61 (Cyr61), a secreted and extracellular matrix-associated signalling protein of the CCN family, has been signalled out as the most regulated gene by Cezanne in VSMCs (*Figure 3A–C*). Moreover, CCN1 has a well-established role in VSMC pathology and neointimal SMC hyperplasia upon vascular injury.^24^ Therefore, as a proof of concept we attempted to investigate how the CCN1 gene expression being regulated by Cezanne in the context of VSMC functions and neointimal formation. Data shown in *Figure 3C* suggested that the deubiquitinating activity of Cezanne is critical for CCN1 gene regulation by Cezanne. To support such a mechanism, western blotting analysis was conducted with total cell lysates and the immunoprecipitated protein by a pan-ubiquitin antibody using anti-CCN1 antibody to examine total and ubiquitinated (modified) CCN1 protein, respectively. We found that while the total CCN1 protein expression level was significantly up-regulated by enforced expression of wild-type but not the mutated version of Cezanne plasmids, the ubiquitinated CCN1 protein was slightly down-regulated by Cezanne overexpression (*Figure 3D and E*), suggesting that Cezanne-mediated protein deubiquitination may play a minor role in stabilizing CNN1 protein.

### 3.6 Cezanne regulates CCN1 through activating β-catenin signalling

**Figure 4:**
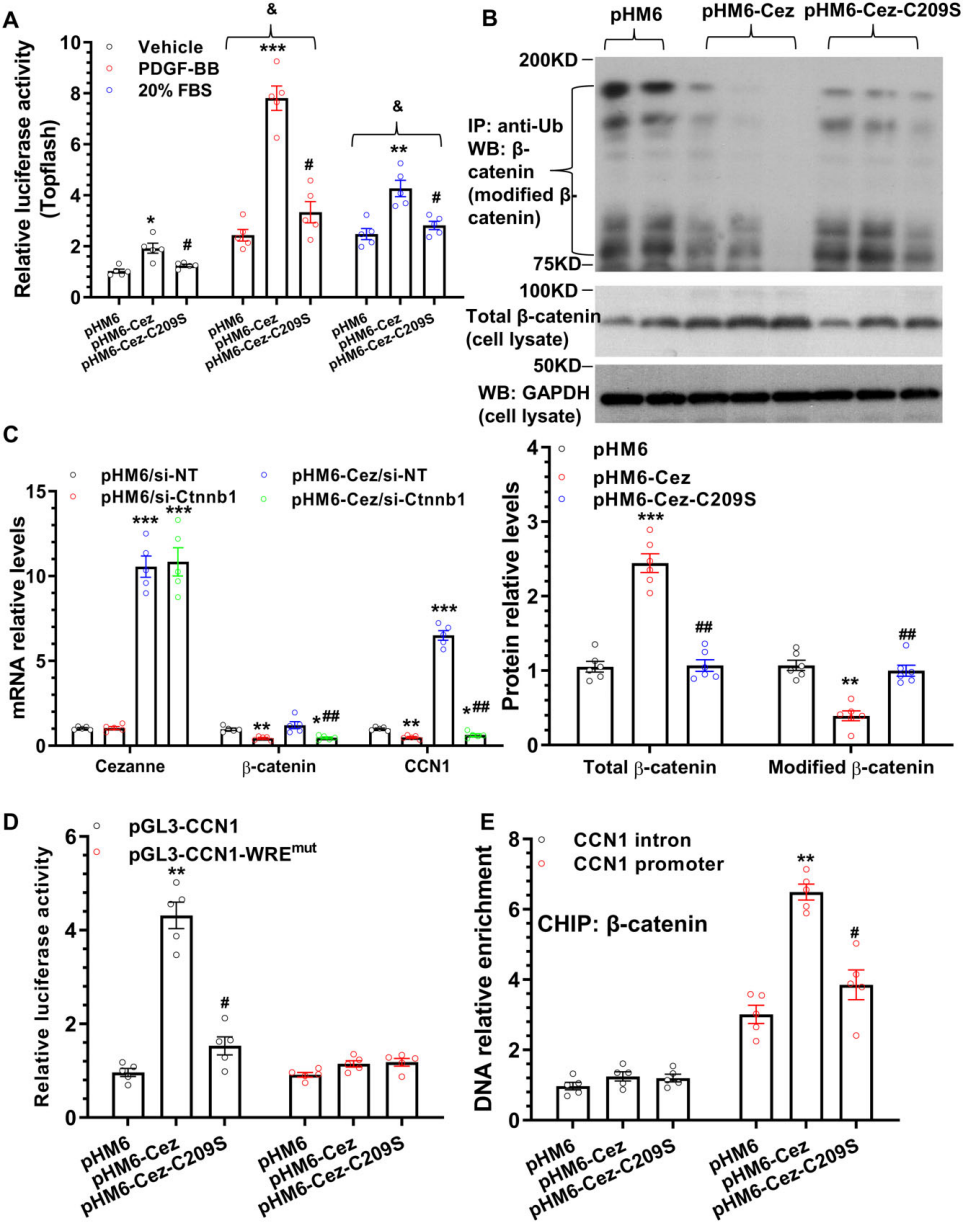
Cezanne regulates CCN1 through activating β-catenin signalling. (*A*) Luciferase activity assays with M50 Super (8x) TOPFlash reporter showed that β-catenin signal pathway was activated by Cezanne over-expression in VSMCs with different treatments. (*B*) Cezanne activates β-catenin signalling by targeting it for deubiquitination in VSMCs. Following similar treatments as described in *Figure 3D*, pan-ubiquitin immunoprecipitates or total cell lysates were prepared to examine ubiquitinated (modified β-catenin) and total β-catenin protein, respectively. (*C*) β-catenin is required for CCN1 gene regulation by Cezanne. VSMCs were co-transfected with pHM6 or pHM6-Cez, and si-NT (non-target or scrambled siRNA) or si-Ctnnb1 (β-catenin specific siRNA) as indicated. Total RNAs were collected and subjected to RT-qPCR analysis. (*D*) Wnt-response element (WRE) within CCN1 gene promoter is required for its gene regulation by Cezanne as demonstrated in luciferase activity assays with wild type (pGL3-CCN1) or WRE mutated (pGL3-CCN1-WRE^mut^) CCN1 gene promoter reporters. (*E*) CHIP assay showed that Cezanne promoted β-catenin binding to CCN1 gene promoter. The data presented here are representative (upper panel in *B*) or mean ± SEM of five (*n* = 5 in *A, C–E*) or six (*n* = 6 in *B*) independent experiments. *P < 0.05, **<0.01, ***<0.001 (vs. pHM6 or pHM6/si-NT); ^#^*P* < 0.05, ^##^<0.01 (pHM6-Cez-C209S vs. pHM6-Cez, or pHM6-Cez/si-Ctnnb1 vs. pHM6-Cez/si-NT); ^&^*P* < 0.05 (vs. vehicle) (one- (*B–E*) or two-way (*A*) ANOVA with a *post hoc* test of Tukey’s analysis).

Previous studies have reported an important role for Wnt/β-catenin signalling in CCN1 gene regulation in cancer^25^ and mesenchymal stem cells.^26^ To address if a similar mechanism is underlying the CCN1 gene regulation in VSMCs, we firstly examined a possible effect of Cezanne on Wnt/β-catenin signalling activity by conducting luciferase activity assays with M50 Super (8x) TOPFlash reporter, and observed that the Wnt/β-catenin signalling activity was expectedly induced by both FBS and PDGF-BB treatments. Importantly, while Cezanne over-expression in VSMCs significantly increased the reporter activity in the absence or presence of the respective stimulations, such induction was blunted by over-expression of the mutated Cezanne (*Figure 4A*), indicating that Cezanne has a regulatory role for Wnt/β-catenin signalling in VSMCs, and its deubiquitinating activity is responsible for such a role. Indeed, an increased level of the total β-catenin, but a decreased level of ubiquitinated β-catenin protein was observed in Cezanne over-expressing VSMCs. Importantly, such regulatory effects were reversed by over-expression of the mutated version of Cezanne (*Figure 4B*). These data have collectively demonstrated that Cezanne up-regulates β-catenin signalling by targeting β-catenin protein for deubiquitination in VSMCs. Moreover, co-transfection experiments were subsequently conducted to examine if there is any potential link among Cezanne, β-catenin, and CCN1 gene expression. Data shown in *Figure 4C* revealed that the β-catenin gene expression level was not regulated by Cezanne overexpression in VSMCs, which was consistent with the RNA sequencing data showing that β-catenin was not among the genes regulated by Cezanne knockdown (Supplementary material online, *Figure S4A*). Importantly, we found that while CCN1 gene expression was significantly down-regulated and up-regulated by β-catenin inhibition and Cezanne over-expression, respectively, CCN1 gene up-regulation by Cezanne was almost abrogated by β-catenin knockdown (*Figure 4C*), indicating that β-catenin is required for Cezanne-mediated CCN1 gene regulation. To further confirm the essence of β-catenin signalling in CCN1 gene regulation by Cezanne, luciferase activity assays were conducted with a wild type or WRE (Wnt-response element) mutated CCN1 gene promoter reporter, respectively. We found that enforced expression of wild type, but not the mutated Cezanne in VSMCs significantly up-regulated CCN1 gene promoter activity, while such regulation disappeared once the WRE within CCN1 gene promoter was mutated (*Figure 4D*). Finally, data from chromatin immunoprecipitation (ChIP) assay showed a significant increased binding of β-catenin to CCN1 gene promoter upon Cezanne over-expression, while such enhancement was almost abolished when overexpressed the mutated version of Cezanne plasmid in VSMCs (*Figure 4E*). We therefore conclude that β-catenin signalling activation and WRE within CCN1 gene promoter were essential for CCN1 gene regulation by Cezanne.

### 3.7 Cezanne regulates VSMC proliferation and migration through modulating CCN1

Co-transfection experiments were conducted to further address the functional implication of CCN1 gene regulation by Cezanne in VSMC pathologies. Gene expression data (Supplementary material online, *Figure S5A*) showed that while CCN1 was successfully down-regulated and significantly up-regulated by CCN1 specific siRNA and Cezanne overexpression plasmid, respectively, Cezanne expression was not affected by CCN1 inhibition, suggesting that CCN1 is indeed the downstream gene of Cezanne. Moreover, β-catenin gene expression was not impaired by either Cezanne over-expression or CCN1 knockdown, further confirming that Cezanne regulates β-catenin at post-transcriptional level. Consequently, we observed that VSMC proliferation was decreased by CCN1 knockdown, but increased by Cezanne over-expression, while the promotive effects of Cezanne over-expression on VSMC proliferation was blunted by CCN1 inhibition in the co-transfecting cells (Supplementary material online, *Figure S5B*). A similar phenomenon was found with VSMC migration (Supplementary material online, *Figure S5C*). These data have collectively showed that CCN1 activation is required for Cezanne-mediated VSMC growth and mobility.

### 3.8 Local Cezanne inhibition in the injured arteries prevented VSMC proliferation and reduced inward arterial remodelling following injury

**Figure 5:**
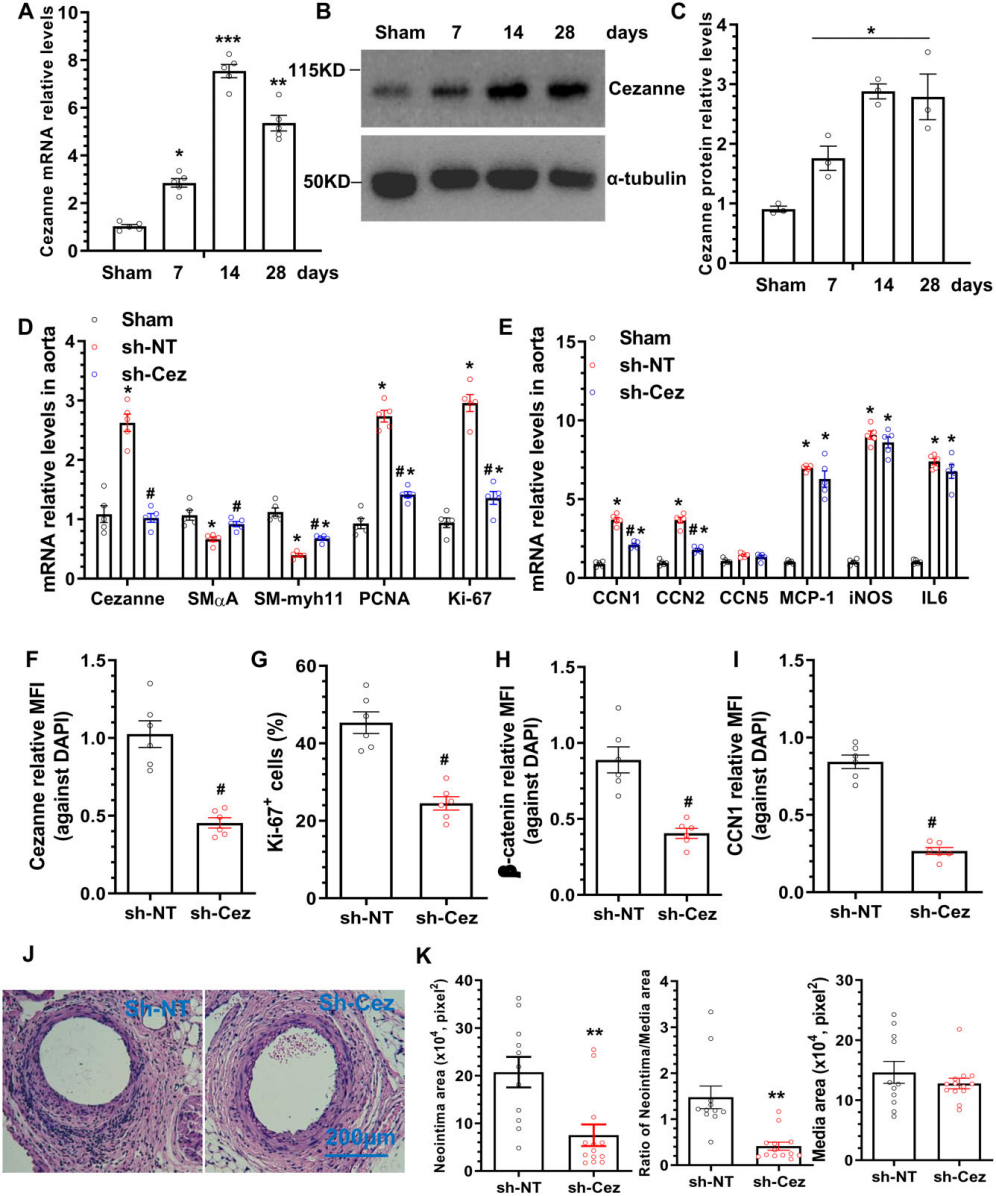
Local restoration of the dysregulated Cezanne expression upon injury reduces neointima formation. (*A–C*) Up-regulated Cezanne expression in response to vascular injury. Injured arterial segments were harvested from mice without (sham) or with injury at the indicated time points and subjected to RT-qPCR (*A*) and western blot (*B* and *C*) analysis, respectively. The data presented in (*A–C*) are mean ± S.E.M. of five (*A*) or three (*B* and *C*) independent experiments (Note: injured arterial segments from 3 or 5 mice were pooled as one independent biological sample in *A* or *B*/*C*, respectively). (*D–E*) Aortic gene expression profiles determined by RT-qPCR analysis. After injury, 10–20 µL of DMEM containing 1.0–2.0 × 10^6^ shRNA lentiviral particles (sh-NT or sh-Cez) was directly infused into the lumen of the injured femoral arteries, followed by a 30-min incubation for local VSMC infection. At seven days (*D–E*), 14 days (*F–I*), or 28 days (*J–K*) post-treatment, injured arterial segments were harvested and subjected to RT-qPCR (*D–E*) and immunofluorescence staining (*F–I*) analyses, respectively. The data presented in (*D–E*) are mean ± SEM. of five independent experiments (injured arterial segments from 3 mice were pooled for one independent experiment). Data presented in (*F–I*) are the quantitative data (mean ± SEM) of relative mean fluorescence intensity (MFI) for Cezanne (*F*), β-catenin (*H*), and CCN1 (*I*) over DAPI, or the percentage of Ki67-positive cells (*G*) in the injured vessels from 6 mice (*n* = 6 mice for each group). *P < 0.05, **<0.01, ***<0.001 (vs. sham); ^#^*P* < 0.05 (sh-Cez vs. sh-NT). (*J–K*) HE staining analysis. Paraffin sections from both groups (*n* = 11 mice for sh-NT, and *n* = 13 mice for sh-Cez) were prepared and subjected to H&E staining analyses. Representative images (*J*) and morphological characteristics (*K*) including neointimal area, neointimal/media ratio, and media area at 28 days post-injury were presented here. **P < 0.01 (sh-Cez vs. sh-NT) (*C*, Kruskal–Wallis test, *A, D, E*, one-way ANOVA with a *post hoc* test of Tukey’s analysis; *F–I* and *K*, unpaired *t*-test).

Immunostaining data showed that Cezanne was strongly expressed in endothelium, but weakly expressed in SMC layer in normal murine aorta (Supplementary material online, *Figure S6*). Moreover, we observed that Cezanne gene and protein expressions were significantly up-regulated upon injury, peaked at 14 days post-injury (*Figure 5A–C*), implying a role for Cezanne in injury-induced arterial remodelling. To investigate the *in vivo* functional relevance of Cezanne in arterial inward remodelling, 10–20 µL of DMEM containing 1.0–2.0 × 10^6^ lentiviral particles (sh-NT or sh-Cez) was directly infused into the lumen of the injured femoral arteries immediately after injury, followed by a 30-min incubation for local VSMC infection. Aortic gene expression data showed that local direct infusion of Cezanne specific (sh-Cez) lentivirus successfully down-regulated Cezanne expression level in the injured vessels (*Figure 5D*). As a result, both cell proliferation genes (PCNA and Ki67) were significantly down-regulated in the injured arteries by sh-Cez (*Figure 5D*), and a similar trend was observed with both CCN1 and CCN2 gene (*Figure 5E*). Interestingly, we found that local Cezanne inhibition increased two SMC genes expressions in the injured arteries (*Figure 5D*). Moreover, we observed no change for CCN5 gene expression in the injured arteries, and dramatic increased expression levels of three inflammatory genes (MCP-1, iNOS and IL6) in the injured vessels, but their expression was not affected by Cezanne inhibition (*Figure 5E*). Consistently, less Cezanne (*Figure 5F* and Supplementary material online, *Figure S7*), β-catenin (*Figure 5H* and Supplementary material online, *Figure S7*) and CCN1 (*Figure 5I* and Supplementary material online, *Figure S7*) protein as well as lower percentage of Ki67-positive cells (*Figure 5G* and Supplementary material online, *Figure S7*) was observed within the media and neointima layers of the injured vessels with Cezanne gene inhibition, confirming that local Cezanne inhibition decreases VSMC proliferation after injury. Importantly, the inward arterial remodelling upon injury was significantly reduced by local restoration of the dysregulated Cezanne expression in the injured arteries (*Figure 5J and K*).

### 3.9 Genetic deletion of Cezanne reduces atherosclerotic lesion size but with increased plaque vulnerability

**Figure 6:**
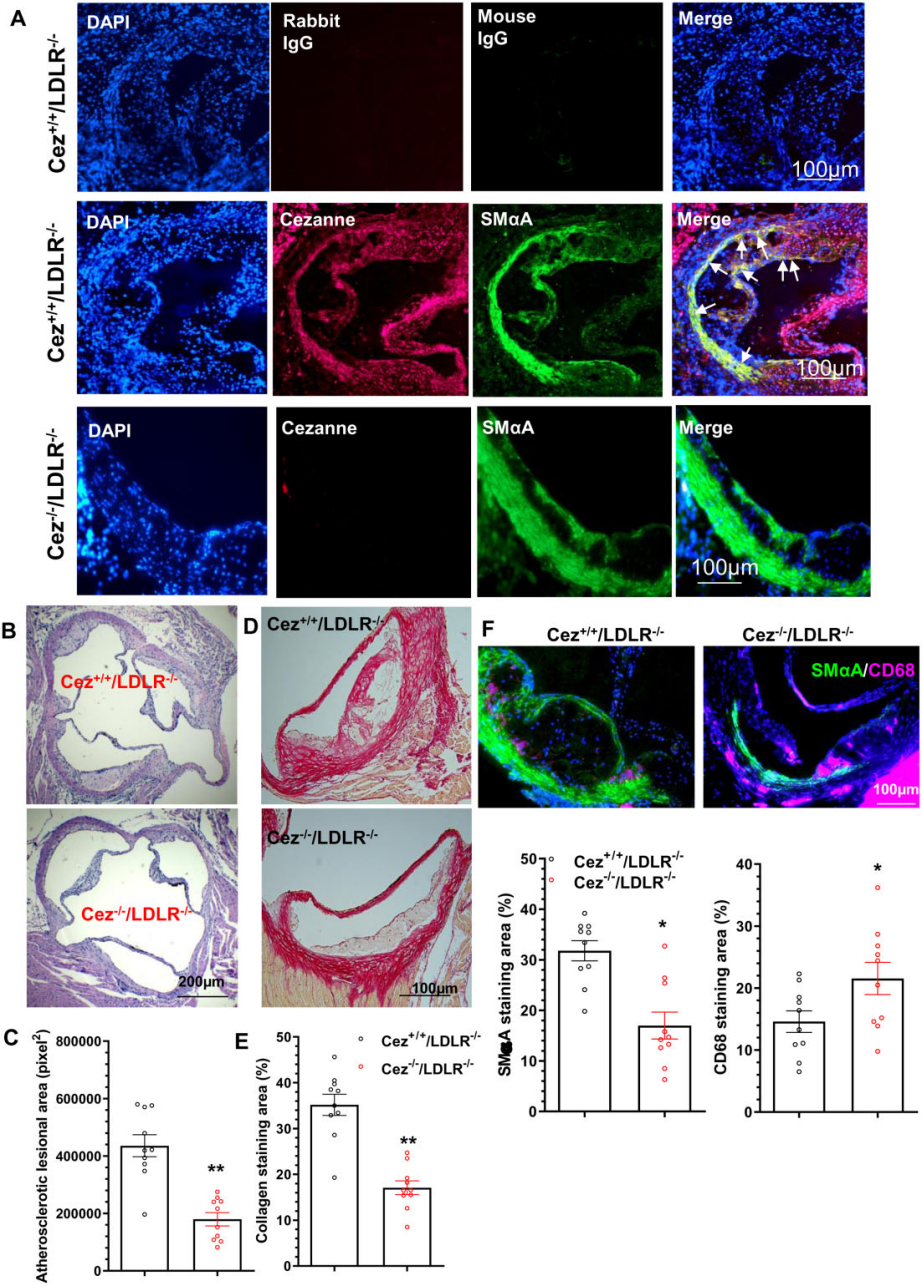
Genetic deletion of Cezanne reduces atherosclerotic lesion size as well as collagen and SMC content. Eight-week-old male Cezanne knockout mice (Cez^−/−^/LDLR^−/−^) and their littermates (Cez^+/+^/LDLR^−/−^) were fed an HFD for 12 weeks. Aortic roots were harvested and subjected to various analysis. (*A*) Cezanne expression in the atherosclerotic plaques. Representative images from six mice (*n* = 6) were presented here. Note: white arrows indicate SMαA^+^ cells co-express Cezanne within atherosclerotic lesion. (*B–C*) HE staining analysis showing Cezanne deficiency resulted in smaller atherosclerotic lesion. (*D–E*) Sirius Red staining analysis showed decreased collagen content within the atherosclerotic plaques of Cezanne knockout mice. (*F*) Immunofluorescent staining showed a lower level of SMCs, but higher level of macrophages in Cezanne knockout mice. Data presented in (*B–F*) are representative (*B, D,* and *upper panel in F*) and the quantitative data (mean ± S.E.M.) of atherosclerotic plaque area (*C*), collagen content (*E*), or staining area for SMA and CD68 (bottom panel in *F*) from 10 mice (*n* = 10 mice for each group) (*C, E*, and bottom panel in *F*, unpaired *t*-test).

Immunostaining analysis showed that Cezanne was expressed in vessel wall of healthy aortic roots, albeit at a low level (Supplementary material online, *Figure S8*). To further explore if Cezanne could play a role in atherogenesis, we crossbred Cezanne transgenic gene-trapped (GT) mice (Cez^GT/GT^ or Cez^−/−^) generated in our previous study^12^ with LDLR^−/−^ mice to generate Cezanne/LDLR double knockout mice (Cez^−/−^/LDLR^−/−^) and their control littermates (Cez^+/+^/LDLR^−/−^). Eight-week-old male mice were fed a high-fat diet to induce atherosclerosis as described in our previous study.^27^ No Cezanne protein expression was detected in atherosclerotic plaques of Cez^−/−^/LDLR^−/−^ mice (*Figure 6A*), validating this genetically modified model. Pathologically, Cez^−/−^/LDLR^−/−^ mice had substantially less aortic atherosclerosis (>55% reduction compared with controls, *P* < 0.001) in aortic roots (*Figure 6B and C*). Importantly, compared to control mice, much less collagen content (*Figure 6D and E*), and a lower level of SMCs, but a higher level of macrophages (*Figure 6F*) were observed in Cez^−/−^/LDLR^−/−^ mice. These observations have collectively confirmed a functional role for Cezanne in atherogenesis and plaque stabilization.

### 3.10 Increased expression of Cezanne in human atherosclerosis

**Figure 7:**
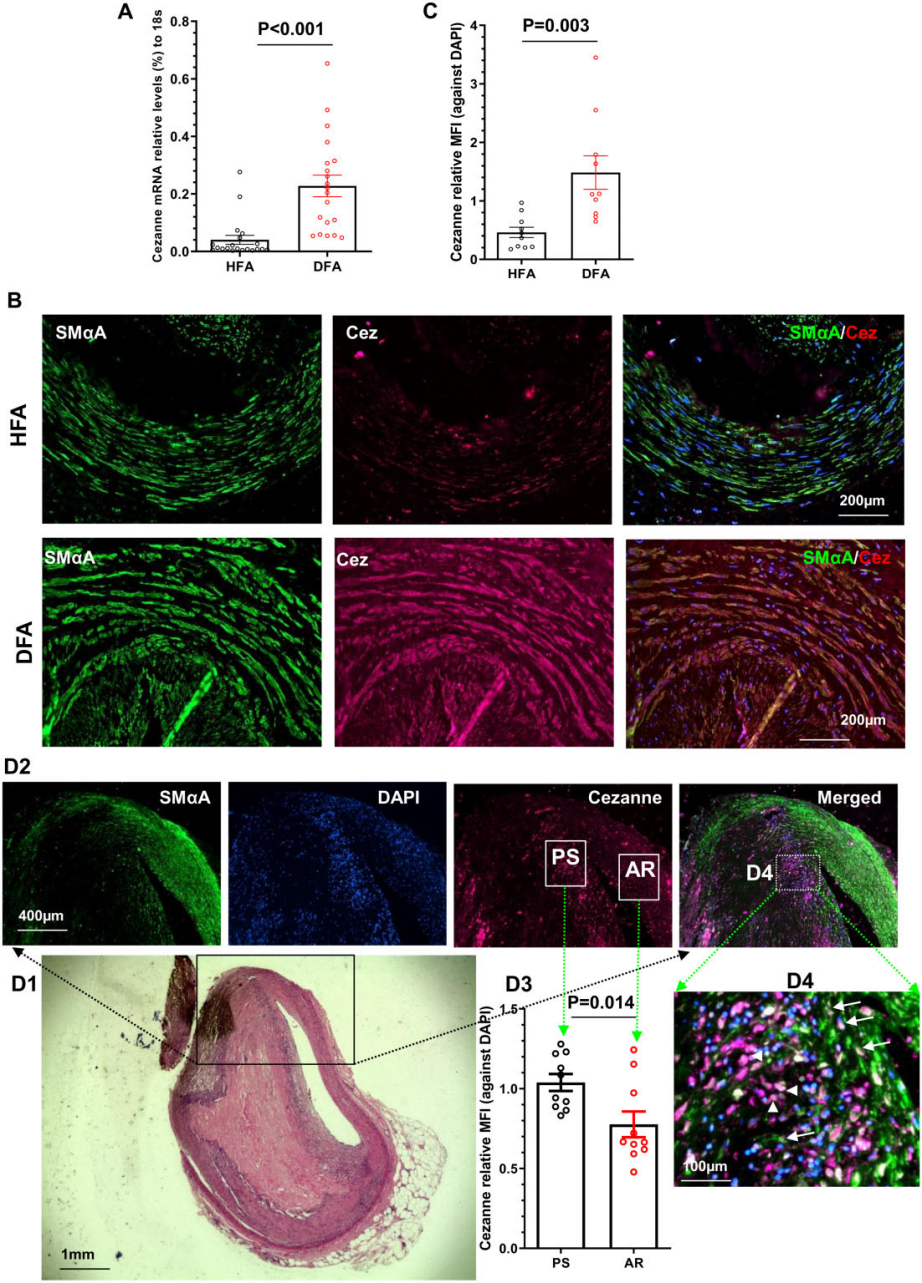
Increased Cezanne expression in the human atherosclerotic plaques. (*A*) RT-qPCR analysis of Cezanne expression in normal healthy (HFA) and diseased human femoral arterial specimens (DFA, *n* = 20). (*B–C*) Increased Cezanne protein levels in diseased femoral arteries as demonstrated in immunofluorescent staining analysis. Representative (*B*) and the quantitative data (mean ± S.E.M.) of relative mean fluorescence intensity (MFI, Cezanne against DAPI) (*C*) from 10 patients (*n* = 10 for each group) were presented here. (*D*) Cezanne detection in human coronary atherosclerotic plaques. Paraffin sections from 10 coronary atherosclerotic plaques (*n* = 10) were prepared and subjected to HE (*D1*) and immunofluorescent (*D2* and *D4*) staining analysis, respectively. Representative (*D1* and *D2*) and the quantitative data (mean ± S.E.M.) of relative mean fluorescence intensity (MFI, Cezanne against DAPI) (*D3*) from plaque shoulder (PS) and adjacent regions (AR) were presented here. Note: arrows and arrowheads in D4 indicate SMαA^+^/Cezanne^+^ and SMαA^−^/Cezanne^+^ cells, respectively. A, C, and D3, unpaired *t*-test.

To translate our findings from mice to men, we first examined a possible role of Cezanne in human aorta SMCs (hAoSMCs). Expectedly, Cezanne over-expression in hAoSMCs (Supplementary material online, *Figure S9A*) significantly increased their proliferation (Supplementary material online, *Figure S9B*) and migration (Supplementary material online, *Figure S9C and D*), while such increase was almost blunted by over-expression of the mutated Cezanne, inferring a similar role for Cezanne in human VSMC functions. Additionally, we demonstrated by RT-qPCR that Cezanne expression is enhanced in human femoral arteries with atherosclerotic lesion compared to those without lesion (*Figure 7A*). Immunofluorescence staining (*Figure 7B and C*) showed a much higher expression level of Cezanne protein in the diseased femoral arterial specimens than that in healthy specimens. Finally, immunofluorescence staining analysis of human coronary atherosclerotic plaques also confirmed a high expression level of Cezanne in coronary atherosclerosis (*Figure 7D*). Importantly, a much higher Cezanne expression level was observed in the plaque shoulder, compared with the adjacent regions resembling normal healthy coronary artery (*Figure 7D2* and *D3*). Taken together, these data demonstrate the potential involvement of Cezanne in human atherogenesis.

## 4. Discussion

Vascular pathologies including atherosclerosis and arterial inward remodelling upon vascular injury remain a challenging issue for treating cardiovascular diseases. Deeper understanding into the aetiology and pathogenesis of vascular pathologies may provide novel therapeutic interventions for various vascular disorders. Here, we document a functional role for Cezanne in modulating VSMC functions and injury-induced vascular remodelling *in vitro* and *in vivo*. We first observed that Cezanne expression was activated by a variety of atherogenic stimuli in VSMCs. With cellular function aspects, we show that Cezanne has a profound role in governing VSMC proliferation and migration, but has a negligible effect on VSMC apoptosis and inflammatory response. Mechanistically, we found that the deubiquitinating activity of Cezanne is critical for its functional effects on VSMC proliferation and migration. Additionally, we have identified that CCN1 is the functional downstream target of Cezanne in the context of VSMC functions and arterial remodelling. We also demonstrate that Cezanne regulates CCN1 expression by activating Wnt/β-catenin signalling through targeting β-catenin protein for deubiquitination. Importantly, we translated our *in vitro* findings to two murine models by demonstrating that vascular injury induced neointima formation is inhibited by local inhibition and restoration of the dysregulated Cezanne expression in injured arteries, and the atherosclerotic lesion is reduced by genetic deletion of Cezanne in a hyperlipidemic mice model. Finally but most importantly, we provide evidence to support the notion that Cezanne may play a role in human atherogenesis.

We have previously reported that Cezanne can be induced in multiple immortalized cell lines (e.g. A549, Hela, and Hek293) by pro-inflammatory stimuli TNFα/IL1,^12^^,^^13^ and in endothelial cells in response to hypoxia-reoxygenation.^12^ In this study, we have shown that Cezanne can also be induced in VSMCs by multiple atherogenic stimuli such as the potent cellular mitogens PDGF-BB and high concentration of serum, and pro-inflammatory stimuli TNFα and Lps. Interestingly, our previous study has demonstrated that hypoxia-reoxygenation induced Cezanne expression in a p38-dependent manner in endothelial cells.^12^ It is plausible that a similar mechanism underlies Cezanne expression induced by abovementioned atherogenic stimuli in VSMCs since it has been extensively reported that p38 mitogen-activated protein kinase signalling can be activated by these stimuli in various cellular contexts including VSMCs.

Cezanne controls cancer cell proliferation, survival, and migration, and is recurrently up-regulated in numerous malignancies.^14^^,^^28^ In this study, we report new cellular functions for Cezanne in regulating VSMC proliferation and migration, and show that the deubiquitinating activity of Cezanne is essential for its promotive effect on VSMC functions. No apparent effects of Cezanne on VSMC apoptosis and TGFβ1-induced VSMC specific genes expression were observed (Supplementary material online, *Figure S2*). However, we found that the expression levels of two SMC genes (SMαA and Myh11) were up-regulated by local Cezanne inhibition in the injured arteries (*Figure 5D*), suggesting a role for Cezanne in SMC gene regulation or SMC differentiation in the context of the injury-induced arterial remodelling. Such a discrepancy is likely due to the fact that there are multiple cellular components in the injured arteries could contribute to the total expression levels of these two SMC genes. It has been widely reported that apart from the medial VSMCs the neointimal SMC-like cells could also be derived from adventitial stem/progenitor cells,^29^^,^^30^ and/or trans-differentiated from either adventitial fibroblasts^31^^,^^32^ or endothelial cells.^33^^,^^34^ Taken together, these data suggest that VSMC functions are selectively regulated by Cezanne, whereas the potential involvements of Cezanne in stem cell differentiation and other cell trans-differentiation in the context of neointimal SMC accumulation remain to be determined.

Opposite to its well-known role in NF-κB signalling and inflammatory response as previously discussed, we surprisingly observed an insignificant role for Cezanne in regulation of VSMC inflammatory response *in vitro* in response to pro-inflammatory cytokines (Supplementary material online, *Figure S3*) and *in vivo* upon vascular injury (*Figure 5E*). These unexpected observations suggest that Cezanne regulates NF-κB signalling and inflammatory response in a cellular context- and/or disease-dependent manner.

Apart from its involvement in cancers, Cezanne has also been implicated in other pathological conditions. It has been reported that Cezanne could inhibit renal inflammation and injury in murine kidneys exposed to ischaemia followed by reperfusion via controlling NF-κB-dependent inflammatory activation.^12^ Cezanne has also been found to regulate excessive endothelial cell proliferation and inflammation at atheroprone regions of arteries via activating hypoxia-inducible factor 1α (HIF1α),^35^ probably also HIF2α.^36^ The later study suggests a role for Cezanne in vascular diseases. This study is the first to report a pathological role for Cezanne in these conditions. By utilising a well-established injury-induced arterial remodelling model, we first demonstrate that local inhibition of Cezanne in the injured arteries significantly prevents arterial inward remodelling. Controlling cell proliferation, not the aortic inflammation is the main contributing factor to the Cezanne-mediated arterial inward remodelling upon injury, which is consistent with the *in vitro* observation. Second murine model, feeding a high-fat diet to the male LDLR^−/−^ mice, was used to further examine the potential role for Cezanne in atherogenesis. We found that atherogenesis in aortic roots was decreased, but plaque vulnerability was increased in Cezanne knockout mice. Reduced SMC content within atherosclerotic plaques observed in Cezanne knockout mice further confirmed a critical role for Cezanne in VSMC proliferation as shown in arterial remodelling model and *in vitro* VSMC functional studies. The clinical relevance of Cezanne in vascular diseases was further confirmed in two distinct human atherosclerotic specimens collected in our previous studies,^17^^,^^22^^,^^23^ femoral arteries isolated from amputated leg of patients with occluded peripheral arterial diseases^17^ and coronary arterial biopsy specimens harvested from patients who died of ischaemic coronary heart disease.^22^^,^^23^ We therefore have collectively demonstrated that Cezanne has a regulatory role in injury-induced arterial inward remodelling and hyperlipidaemia-induced atherogenesis, supporting its therapeutic potential in various vascular diseases including post-angioplasty restenosis and atherosclerosis. However, we should be cautious when considering any Cezanne-modifying agent for treating atherosclerosis since Cezanne inhibition could potentially increase plaque vulnerability to rupture, and therefore further studies using plaque rupture models, in mice or large animals, are warranted.

We have originally speculated that modulating VSMC inflammatory response could be the key underlying mechanism of Cezanne-mediated VSMC functions and arterial inward remodelling, but failed to confirm it (Supplementary material online, *Figure S3*). Alternatively, we conducted the unbiased RNA sequencing analysis to uncover the whole transcription profile modulated by Cezanne. To avoid any potential artefactual effect of the enforced expression of Cezanne in VSMCs on the transcription profile, total RNAs isolated from VSMCs with Cezanne knockdown were chosen for RNA sequencing analysis. We found a relative small number of genes (75 genes) were modulated by inhibiting the endogenous Cezanne expression level in VSMCs stimulated by TNFα. GO term enrichment analysis revealed that Cezanne-mediated genes are mainly associated with cellular and protein bindings (e.g. CCN1/Cyr61, CCN2/Ctgf, CCN5/Wisp2, Ly96, and Shroom3), cellular protein modification process (e.g. Inhbb, Loxl4, Ptpn12, Lox, Gpaa1, Usp50, and Zdhhc7), supramolecular fibre and actin cytoskeleton organization (e.g. Limk1, Loxl4, Lox, Shroom3 and Amotl2) and cell migration/adhesion (e.g. Amotl2, Sema3f, CCN1/Cyr61, CCN2/Ctgf, CCN5/Wisp2, and Fbln2), which further support our observations that Cezanne specifically controls VSMC growth and mobility. Among the 58 genes down-regulated by Cezanne inhibition (Supplementary material online, *Figure S4A*), the extracellular matrix-associated signalling protein CCN1 caught our attention due to its reported cellular functions in VSMC senescence,^37^ proliferation,^38^ migration,^39^ and adhesion,^40^ and its potential implications in neointima formation,^24^^,^^38^^,^^41^ arteriosclerotic intimal hyperplasia,^42^ as well as atherosclerosis.^43^^,^^44^ All of these observations make CCN1 a perfect downstream target of Cezanne signalling in the context of VSMC pathology and vascular diseases. Indeed, as shown in *Figure 3A*. CNN1 is the most significant down-regulating gene in response to Cezanne knockdown in VSMCs. Functionally, we show that CCN1 gene activation is required for Cezanne-mediated VSMC functions since simultaneously over-expressing Cezanne and knocking down CCN1 in VSMCs almost blunted the promotive effects of Cezanne over-expression on VSMC proliferation and migration (Supplementary material online, *Figure S5*).

However, how does Cezanne regulate CCN1 gene and protein expression in VSMCs? Previous studies have shown that Cezanne can direct bind to ubiquitin,^45^ particularly the Lys63- and Lys11-linked polyubiquitin chains,^46–48^ and remove those polyubiquitin chains from signalling intermediaries to regulates distinct cellular functions.^12^^,^^13^ Our data showed that the deubiquitinating activity of Cezanne is required for both Cezanne-mediated CCN1 gene and protein up-regulation, indicating that Cezanne regulates CCN1 at transcriptional level, or via a transcriptional mechanism. Indeed, immunoprecipitation assay using a pan-ubiquitin antibody revealed that CCN1 protein ubiquitination was not controlled by Cezanne. We therefore speculated there is another protein functioning as the signalling intermediary between Cezanne and CCN1 regulation, such as β-catenin. The rationale behind the choosing of β-catenin due to following facts: emerging evidence in the past decade has not only established a critical role for Wnt/β-catenin signalling pathway in embryogenesis and development, but also suggested that they play a part in cardiovascular disorders through regulating VSMC proliferation, migration, and survival.^49^ Importantly, β-catenin has also been identified as the key regulator in vascular endothelium denudation-induced neointima formation and progression in our previous studies.^50^^,^^51^ The respective relevance and significance of Wnt/β-catenin signalling in VSMC functions and vascular remodelling has been further delineated *in vivo* using an inducible, conditional genetic deletion of β-catenin in SMCs of adult mice.^52^ Although this pathway showed an undoubted role in VSMC physiology and vascular diseases, our understanding of the underlying mechanisms how this pathway to be regulated in vascular disorders, however, remains incomplete. In this aspect, we have now established a mechanistic link between Cezanne, Wnt/β-catenin signalling pathway, and CCN1 regulation in the context of VSMC functions and vascular diseases (*Figure 4*). Specifically, we provide comprehensive evidence to show that Cezanne can directly target β-catenin protein for deubiquitination, which in turn stabilize β-catenin protein, activating Wnt/β-catenin signalling pathway. Further data from co-transfection (Cezanne over-expression and β-catenin knockdown) experiments, CCN1 gene promoter activity assay, and CHIP assays confirmed the essence and necessity of β-catenin and its DNA binding element WRE within CCN1 gene promoter in CCN1 regulation by Cezanne.

One limitation in this study is that atherosclerosis was studied using Cezanne knockout mice, whereas a well-established shRNA lentiviral gene transfer technique was used to evaluate the possible functional role of Cezanne in VSMC functions and injury-induced arterial remodelling. This disparity was because we could not transfer knockout mice between institutes due to technical and regulatory issues. An additional limitation is that Cezanne global knockout mice were used in this study, therefore the observed effects of Cezanne on atherogenesis could also attribute to any potential effects of Cezanne on other cells such as endothelial cells and inflammatory cells, as evidenced by that Cezanne has been previously demonstrated to play important roles in endothelial cells,^12^ and it is highly expressed in endothelium (Supplementary material online, *Figure S6*) as well as SMαA^−^ cells within atherosclerotic plaques (*Figure 7D4*). Despite these limitations, we have conclusively demonstrated that Cezanne is a functional modulator for VSMC growth and mobility, injury-triggered arterial inward remodelling, and hyperlipidemia-induced atherogenesis. These functional and mechanistic observations could open a new avenue to further explore their therapeutic potential for treating post-angioplasty restenosis and atherosclerosis by targeting Cezanne/β-catenin/CCN1 signalling axis.

## Supplementary material

Supplementary material is available at *Cardiovascular Research* online.

## Authors' contributions

W.A. carried out the majority of cellular and molecular genetic studies, immunostaining, and data analysis. L.A.L. carried out some in vitro cellular studies. N.P.B. carried out atherosclerosis work. M.Y., W.W., and X.Z. helped to conduct the animal experiments and human aortic gene analysis. C.L., K.N., and J.L. carried out the immunoassays. C.Z. and X.S. helped to perform hAoSMC works. R.P. provided human coronary arterial samples and analysis. L.Z. helped to draft the manuscript and critical reviewing. P.C.E. and Q.X. conceived of the study, and participated in its design and coordination and helped to draft the manuscript. All authors read and approved the final manuscript.

**Conflict of interest:** none declared. 

## Funding

This work was supported by British Heart Foundation (PG/15/11/31279, PG/15/86/31723, PG/16/1/31892, PG/20/10458, and RG/13/1/30042). This work forms part of the research portfolio for the National Institute for Health Research Biomedical Research Centre at Barts.

### Data availability

Some data may not be made available because of privacy or ethical restrictions. All remaining data are contained within the article, and the data that support the findings of this study are available from the corresponding author on reasonable request.

## Supplementary Material

cvab056_Supplementary_Data

## Notes

Translational Perspective In this study, we have identified the deubiquitinating enzyme Cezanne as a novel regulator in governing VSMC phenotype, injury-induced neointimal hyperplasia, and hyperlipidaemia-induced atherosclerosis. Since accumulating evidence highlights an important role for VSMC dysfunctions in many cardiovascular pathological conditions including atherosclerosis, arterial remodelling, hypertension, and stroke, local modulation of this newly identified signal axis (Cezanne/β-catenin/CCN1) could represent as a novel therapy for post-angioplasty restenosis and aforementioned diseases.
