# Supplementary material for: Cezanne is a critical regulator of pathological arterial remodelling by targeting β-catenin signalling
Source: Cardiovasc Res. 2021 Feb 18;118(2):638–53. doi: 10.1093/cvr/cvab056 (PMC8803089; doi:10.1093/cvr/cvab056)

Supplementary Data

Title: Cezanne is a critical regulator of pathological arterial remodelling by targeting β-catenin signalling

Running Title: Cezanne in vascular diseases

Weiwei An, et al.

Detailed Methods and Materials

Materials. Antibodies against Cezanne/OTUD7B (Rabbit IgG, 16605-1-AP) was purchased from Thermo Fisher Scientific (UK), and CCN1/Cyr61 (Rabbit IgG, bs-1290R) was from Stratech Scientific Ltd (UK), respectively. Antibodies against Ubiquitin (Rabbit IgG, ab7780; for immunoprecipitation (IP) assays), β-catenin (Rabbit IgG, ab32572), Ki67 (Rabbit IgG, ab15580), CD68 (Rabbit IgG, ab125212), and GAPDH (mouse IgG, 6C5, ab8245) were purchased from Abcam (Cambridge, UK). Antibodies against α-tubulin (mouse, T6074), and SMαA (mouse, 1A4, A5228) were from Sigma (USA). All secondary antibodies were from Thermo Fisher Scientific (UK). Other materials used in this study were purchased from Sigma unless specifically indicated.

VSMC culture and treatments

Mice used for VSMC isolation were euthanized by placing them under deep anaesthesia with 100% O2/5% isoflurane, followed by decapitation. Primary murine VSMCs were isolated from aorta (ascending to abdominal aorta) of eight-week old C57BL/6 mice with both sexes, and routinely maintained in DMEM supplemented with 10% FBS as described in our previous studies[1-6]. As previously described, VSMCs between passages 3 (P3) to 8 were used in the current study since the gene expression levels of VSMC contractile markers could maintain at a comparable level in the cultured VSMC with early passages (up to passage 8)[4]. Human aortic SMCs (hAoSMCs) were purchased from PromoCell GmbH (C-12533) and cultured in SMC growth medium 2 (PromoCell GmbH, C-22062) according to the manufacturer's instructions. Both murine VSMCs and hAoSMCs between P3 and P8 were treated with various atherogenic stimuli as described in the previous studies[1-6]. Briefly, VSMCs were serum starved for 24~48 hours (0% FBS), followed by an incubation with different atherogenic stimuli as indicated for up to 48 hours.

Gene over-expression plasmids and/or MISSION esiRNA transfection

For Cezanne plasmid transfection, control (pHM6), wild-type (pHM6-Cez), or mutated Cezanne (pHM6-Cez-C209S) plasmids generated in our previous study[7] were transfected into VSMCs (1.0µg per 10^6 VSMCs) using TurboFect Transfection Reagent (Thermo Fisher Scientific Inc) according to the manufacturer's instructions.

For plasmid and siRNA co-transfection, respective control or gene over-expression plasmids (1.0µg per 10^6 VSMCs), and/or non-target (si-NT) or gene-specific siRNAs (si-CCN1 or si-Ctnnb1) (50 nM, final concentration) were co-transfected into VSMCs using TransIT-X2 Transfection Reagent (Geneflow Limited, UK) according to the manufacturer's instructions, and

described in our previous study[3]. Briefly, VSMCs (1.5~2.0 x 10^5 per well) were seeded into six-well plate 24 hours prior to transfection. Ten µl of siRNAs (10µM in stock) and appropriate amount of respective plasmid (200ng of plasmid) were mixed with 250µl of serum free DMEM in a sterile Eppendorf tube, followed by adding 7.5µl of TransIT-X2 reagent. After incubated at RT for 30 mins to allow the complexes to form. The TransIT-X2/plasmids/siRNAs complexes were added dropwise in circular motions to ensure all the cells being covered by the mixture. The transfected cells were cultured overnight prior to medium change for serum starvation. MISSION esiRNA are a heterogeneous mixture of siRNAs that all target the same mRNA sequence, resulting in a highly specific and effective gene silencing. All siRNAs (EHUEGFP for si-NT, EMU026621 for si-CCN1, and EMU047621 for si-Ctnnb1) were purchased from Sigma. With an optimum condition (clean and healthy VSMCs at exponential growth phase with 50~70% confluent), a satisfactory transfection efficiency (>60%) is normally achieved with primary VSMCs in our Laboratory using the transfection protocols.

Generation of mouse CCN1 gene promoter reporter and the WRE mutant

The DNA sequence of murine CCN1 gene promoter shown below was synthesized by Genscript Biotech (Nanjing, China), and sub-cloned into the Kpn I and Mlu I sites of the pGL3-basic vector (Promega), designated as pGL3-CCN1. Wnt response element (WRE) mutations were introduced into pGL3-CCN1 by using QuikChange™ site-directed mutagenesis kit (Agilent Technologies) and their respective mutant primers (**Table S1**), according to the manufacturer's instructions, designated as pGL3-CCN1-WRE^{mut}. The resultant vectors were verified by DNA sequencing.

>mm9_dna range=chr3:145312925-145314471
GGGCGCTCTCGCTCACGGTCTGCCCCAGCGCCGGGAGCCCGCCCTTTA
TAATGCCTGCCTAGGCGCCGCGTGTTGCAGTGACGTAGCTCTGTCGGC
GCGTTCTAGAATTCTCAAACATCTCAGGAATGTTGGTTGGCGCGGGCTG
CTGCCAAGCACCTCCCCTGGCTCCGTTGCACCTTTTCCCCCCTCTTCTCA
GATCCATCAGAGAGAAAAAAAAGTTCGTTTTTTTCCACTTTAAATAACAAC
TTCCTTTGGGGAGGGCGGGATGCTTGGGGCGCGAGGGTGATGATTTTG
ATTGAAGAGGGTATATGGGGGGGGGGGGCGTGGTGTATGTGTTGGTGT
GAACCCTTCAGATCTTTGCCTAGACATGGGAGGGCGGGAGACAGAGGA
ACAAAAGTGGTTTTGTTTGGTGATGCCTACCGGGAAAGGCAGCGAGCTG
TTTGCTTGTTTACAACCAAGGTGAGAGTAAAGTTATCTACTGAGAGTCCA
AAGGGGAGGGGGAGTTGGTTTCTAAGCCAGGGATCTATTTGTGGACTTC
AAAGAGGCATGCTCGGATCAAGTCCCAAAGCTTTGGCATGATGTTTATTA
GCCAAGAGAAGTGTATTGCAAATTAATACCCGGAGTGGCATTTATGAATG
GGGGGGGGGAGGGAAGCTTCCCTCTTCGCTGGCATATTTCCAGCCAGA
GGTCTTCCCTCATCCCTCCCCCTTTTTTTTGGTATGTTGTACTTGTTTGTT
TTTGGAGGTTCCCAGTTTTAGTTCCAGCCCACTGCCTGCATTGGAAATCC
AAGAGGAAACGAGTGCAGAGTTCTCCCGGAGCCTGACGTCCACTTCCTC
ACGGATGCCGGAGGCCGGGTCAACCCTCCTGGCCTCTCTCCCTGGACT
GGCGGCGGTGGAAACTGAGCAGCCAAAGCAGAAGCGGAGGAGACCAC
CGTGGAGTGACTTGTCCTGAAATCACCAGGTGCAAAAGCTACCGGGACA
AAACAGATTCTAACAGGACCATTGTGCCCTGTGGATAGCTGGAAAAAAG
ACTCGGGGTGTTTGCAGACACCCTCCCCCACCGCCCCCTATGTGTCTCG
CTGTGTGTATTTGTTTGCCCCCCCCCCGTGTGTGTGAGTGTGTGTGTGT

GTGTGTGTGTGTGTGTGTGTGTGTGTCTGGGCCCGTCCATGTGTTTAACT**CCTTTGTT**TTGGATCACTTAGAAGCCTTCTTTTTCAAGGGTAGTGCAGTGGCTCCCTCCAAGTTCCGAGACATGAGGGATTTAGAAGGCAACACTGTAATAGAGGAAGTTGGGTTTTAAGATCTTATTAAACATACAACTGAAGACACCCCTCCCACACAAGACCCTATTTGGGGTTATTAATGCCATTCATAAACTGGGATAGCTTTTCTTCATTTTTAATATTTAGGGCCTCTCTGCCTTTGCAGCTTCGCGATCTTGCTTAAGAGAGCCTCCTGCGTTGCCTGTTCTATGAATGACTCCAGTGAGAAGGAGAGGCTGTTGCGCACACTCCAGTGAAGACAGCTGTTACTGGAAAGAGTTAAGTTCATTC

Note: Wnt-response element (**CCTTTGTT**).

Reporter transient transfection and luciferase activity assays

Gene reporter plasmids transfection and luciferase assays were conducted as previously reported[1, 8-10]. Briefly, VSMCs were cultured in 24–well plates overnight and co-transfected with respective reporter plasmids (pGL3-CCN1, pGL3-CCN1-WRE^{mut}, M50 Super 8x TOPFlash[6], or pGL4-NF-κB[6], 0.15 μg/well) and Cezanne over-expression plasmids (pHM6, pHM6-Cez, or pHM6-Cez-C209S, 0.15 μg/well), using TurboFect Transfection Reagent (Thermo Fisher Scientific Inc), according to the manufacturer's instructions. pTK-Renilla (20ng/well) was included in all transfection assays as internal control. Dual-luciferase activity assays were conducted 48 hours after transfection using a standard protocol. Relative luciferase unit (RLU) was defined as the ratio of Luciferase versus Renilla activity with that of the control (set as 1.0).

Cezanne shRNA lentivirus generation and infection

Cezanne shRNA lentiviral particles were generated as described previously[4, 11]. Cezanne shRNA lentiviral particles were produced using MISSION shRNA Otud7b plasmids DNA (SHCLNG-NM_001025614, MISSION® shRNA Bacterial Glycerol Stock, Sigma) according to protocol provided. The shRNA Non-Target control vector (SHC002) was used as a negative control (sh-NT). Briefly, 293T cells were transfected with the lentiviral vector and the packaging plasmids, pCMV-dR8.2 and pCMV-VSV-G (both obtained from Addgene), using TurboFect Transfection Reagent (Thermo Fisher Scientific Inc) according to the manufacturer's instructions. The supernatant containing the lentivirus was harvested 48 hours later, filtered, aliquoted, and stored at –80°C. shRNA lentiviral infection and Cezanne stable knockdown VSMC generation were performed as described in our previous studies with some modifications[4, 11-13]. Briefly, VSMCs were plated 24 hours prior to infection in 6 well-plates at 37°C. One transducing Unit per cell (or 2-3x10^5/well) of sh-NT or sh-Cezanne lentivirus were added with 10μg/ml hexadimethrine bromide (H9268; Sigma). After incubated for 40~48 hours, the media was replaced with complete media containing 4μg/ml puromycin (P9620, Sigma). For selection of transductants, fresh media containing puromycin was added at 2-3 day intervals for 10 days. Stably infected cells with the highest knockdown efficiency were used for functional analysis.

VSMC proliferation assays: manually cell counting & BrdU incorporation assay

As described previously[1-6], VSMCs ($5x10^4$ per well) cultured in 12 well plates were transfected or infected with respective plasmids/siRNAs or shRNA lentivirus as indicated in the each figures. After cultured overnight, the cells were starved by culturing them in the serum-free DMEM supplemented for further 48 hours, followed by 20%FBS or PDGF-BB (10ng/ml) stimulation for additional 48 hours before trypsinizing and manually counting the cells under hematocytometer. For BrdU incorporation assay, VSMCs were transfected with plasmids/siRNAs or infected with lentiviral particles as indicated in each figure, and were re-cultured (0.75 $x10^4$ per well) in 96 well plates overnight, followed by serum starvation for 48 hours. Starved VSMCs were re-stimulated with 20% FBS or 10ng/ml PDGF-BB, respectively, for 48 hours. Cell proliferations were evaluated using 5-Bromo-2'-deoxy-uridine (BrdU) Labeling and Detection Kit II (Roche) according to the manufacturer's instructions. The absorbance of the samples was measured by a microplate reader at 405nm (OD405) with reference measurement at 490nm (OD490). Relative Absorbance (A_{405nm}-A_{490nm}) values representing cell proliferation ability were compared between treatments with control sample set as 1.0.

VSMC Trans-well migration assay

Similar to our previous studies[1-6], VSMCs transfected or infected with respective plasmids/siRNAs or shRNA lentivirus as indicated in the each figures were cultured in serum-free DMEM for 48 hours, and harvested for counting. An aliquot (250,000 cells/200µl) of the cells in serum-free DMEM was dispensed into the trans-well inserts (8µm pore size, Greiner Bio-One Ltd, UK. Item number: 662638) pre-coated with 0.5% gelatin (Sigma, G1393), and DMEM with 20% FBS or 30ng/ml PDGF-BB was placed in the lower chamber. The trans-well plates were incubated at 37°C in a 5% CO2 incubator for 12~18 hours. Non-migrated cells in the top insert were carefully removed by cotton swab, and the migrated cells in the bottom side were stained with Crystal Violet dye. Images were captured at five fixed locations (right, bottom, left, up and centre), and migrated cells were counted by two experienced investigators blinded to the treatments.

VSMC apoptosis (TUNEL) analyses

As reported previously[6], Terminal deoxynucleotidyl transferase dUTP nick end labeling (TUNEL) Assay Kit (11684795910, Sigma) was used to assess VSMC apoptosis by close following the manufacturer's instructions. After staining, images were randomly taken with GFP (Green) and DAPI (Blue) channel, respectively, and pseudo images were created using EVOS FL Auto Imaging System (Thermo Fisher Scientific, UK). TUNEL-positive cells over total cells were counted by two experienced investigators blinded to the treatments.

Immunoblotting

Equal amount of protein was separated by SDS-PAGE with 4%~20% Tris-Glycine gel (Invitrogen, Carlsbad, CA, USA) and subjected to standard Western blot analysis. The blots were subjected to densitometric analysis with Image J software. Relative protein expression level was defined as the ratio of

target protein expression level to α-tubulin or GAPDH expression level with that of the control sample set as 1.0.

Real time quantitative PCR (RT-qPCR) analysis

RT-qPCR was performed as previously described[8-10]. Briefly, total RNAs were isolated from cells using TRI reagent (Sigma) according to the manufacturer's instructions, and subjected to DNase I (Sigma) digestion to remove potential DNA contamination. Reverse transcription was performed using an Improm-II™ RT kit (Promega, Madison, WI, USA) with RNase inhibitor (Promega), and Random primers (Promega). The resultant cDNA was diluted to a working concentration of 5ng/μl and stored at -20ºC for future using. Relative mRNA expression level was defined as the ratio of target gene expression level to 18S expression level, respectively, with that of the control sample set as 1.0. Primers were designed using Primer3-BLAST (National Center for Biotechnology Information, USA) and the sequence for each primer was listed in **supplementary Table S1**.

Immunoprecipitation (IP) assays. VSMCs transfected with control or respective Cezanne over-expression plasmids were washed and harvested in ice-cold lysis buffer (10^7 Cells/ml). After lysed and centrifuged in a microcentrifuge at 4°C for 20 minutes, the supernatant was carefully collected and placed in a fresh tube kept on ice. Equal amount of samples (40–50 μg) were diluted into 1 ml immunoprecipitation buffer, and incubated with 2.5μg anti-Ubiquitin (Rabbit IgG, ab7780) antibody, or equal amount of rabbit IgG at 4°C overnight under gentle rotation. After then, 70~100μL of the protein A-coupled Sepharose beads were added into each sample, and incubated at 4°C for 4 hours under gentle rotation. After washed the beads with washing buffer three times, the immunoprecipitates were eluted from the beads using 2 x SDS loading buffer, and subjected to standard Western blot analysis.

Chromatin immunoprecipitation (ChIP) assay. The ChIP assays were performed as described in our previous studies[4, 6]. VSMCs transfected with pHM6, pHM6-Cez or pHM6-Cez-C209S were treated with 1% (v/v) formaldehyde at room temperature for 10 min and then quenched with glycine at room temperature. The medium was removed, and cells were harvested and sonicated. The sheared samples were diluted into 1 ml immunoprecipitation buffer containing 25 mM Tris-HCl, pH 7.2, 0.1% NP-40, 150 mM NaCl, 1 mM EDTA, and immunoprecipitation was conducted with 5μg antibody raised against β-catenin (Rabbit IgG, ab32572), together with single-strand salmon sperm DNA saturated with protein-G-Sepharose beads. Normal rabbit IgG was used as a control. The immunoprecipitates were eluted from the beads using 100 μl elution buffer (50 mM NaHCO3, 1% SDS). A total of 200 μl proteinase K solution was added to a total elution volume of 300 μl and incubated at 60°C overnight. Immunoprecipitaed DNA was extracted, purified, and then used to amplify target DNA sequences by RT-qPCR. Promoter DNA enrichment with specific antibody was calculated using percent input method with that of the IgG control set as 1.0. The relative level of promoter DNA enrichment was defined as the ratio of promoter DNA enrichments in the samples with treatment(s) (pHM6-Cez or oHM6-Cez-C209S) to the control samples (pHM6) with that of the control sample set as

1.0. PCR amplification of the murine CCN1 gene intron-1 regions were included as additional control for specific promoter DNA enrichment.

RNA sequencing and data analysis

Total RNA was extracted from samples using TRI Reagent® solution from Sigma, and mRNA was purified from total RNA using oligo (dT) magnetic beads. RNA quality control and cDNA library preparation was performed at our in house Genome Centre at Queen Mary University of London (http://www.smd.qmul.ac.uk/gc/Services/SeqRNA/index.html). The cDNA library quality was determined on the Agilent Bioanalyzer 2100 system, followed by sequencing on Illumina NextSeq 2000 system. Original image data generated from NextSeq was transferred into sequencing reads through base calling, and defined as raw reads. Partek® Flow® pipeline was used for sequencing data analysis. Briefly, Adaptor sequences and low-quality sequences were filtered out, and the remaining reads were mapped to the mouse genome mm10 using STAR -2.6.1d, and no more than 2 mismatches were allowed during the mapping read procedure. The gene expression levels were normalized and presented as RPKM, which were used for comparing the difference of gene expression among different treatments. Panther Gene ontology (GO)-Slim analysis and Overrepresentation Test (with the Bonferroni correction for multiple testing) were used for GO enrichment analysis of differently expressed genes. The fold change was transformed using the $\log_2$ function, so the data are centred around zero, whereas the *P* value was $-\log_{10}$ transformed for volcano plot analysis.

Morphometric analysis & quantification of neointimal lesion

Similar to our previous studies[1-6], the injured arteries (~5.0mm from injury site) were harvested, fixed in 4% formaldehyde, and proceed for microtoming. Sections (8µm) were collected at 200µm intervals, mounted on slides, and numbered. Five digitised sections with same identification number from five segments/intervals of each animal were stained with H&E for lesion quantification as previously described[1-6], using a computerized image analysis system ($pixel^2$, Axiovision software) by two experienced investigators blinded to the treatments.

Tissue immunofluorescence staining

Three paraffin sections from same segment/interval of each animal were deparaffined with xylene and rehydrated with ethanol, and then incubated with Tris-EDTA Buffer (10mM Tris Base, 1mM EDTA Solution, 0.05% Tween 20, pH 9.0) at 95~100˚C for 30 minutes to retrieve antigens, followed by incubation with 10% FBS in PBST (PBS plus 0.05% Tween 20) for 30 minutes. Thereafter, the sections were incubated with indicated primary antibodies or respective IgG controls diluted in blocking buffer at 4°C overnight. After washed, sections were incubated with an appropriate Alexa Fluor Plus 488/594/647-conjugated 2nd antibody (ThermoFisher UK, 1:1000 dilution), followed by nuclei staining with 4,6-diamidino-2-phenylindole (DAPI) (1ug/ml). After mounting, the slides were examined using a laser scanning confocal microscope (Zeiss LSM 510 Mark 4) and Zen 2009 image software. The mean fluorescence intensity (MFI) for red (Cezanne, β-catenin and CNN1) and blue (DAPI) fluorescence signal of the selected regions (media and

neointima layers, excluding endothelium of murine aortas) from each aortic section were measured with Image J pro software by two experienced investigators blinded to the treatments, and presented as the relative MFI (target proteins over DAPI). Three sections were analyzed per vessel or aortic roots, and averaged.

Atherosclerosis and Characterization

Cezanne transgenic gene-trapped (GT) ($Cez^{GT/GT}$ or $Cez^{-/-}$, C57BL/6 background) mice used in our previous study[14] were crossbred with $LDLR^{-/-}$ mice (C57BL/6 background, bred in house) to generate $Cez^{+/-}$/$LDLR^{+/-}$ double heterozygous mice. $Cez^{+/-}$/$LDLR^{+/-}$ double heterozygous mice were bred to produce $Cez^{-/-}$/$LDLR^{-/-}$ double knockout mice and their control littermates ($Cez^{+/+}$/$LDLR^{-/-}$). Eight-week-old male mice were fed a high-fat diet (HFD) containing 21% fat, 1.25% cholesterol, and 0% cholate (AIN-76A/Clinton-Cybulsky Cholesterol Series #3-108, T-58R6-1810021, Test Diet Limited) for 12 weeks to induce atherosclerosis as described in our previous study[15]. At the end of protocol, the heart harbouring the aortic roots was carefully isolated and cut from the level above the coronary artery at the base of the heart. The hearts were fixed in 4% formaldehyde, and proceed for paraffin embedding and microtoming. The extent of atherosclerotic lesions of aortic roots, and collagen content within atherosclerotic lesions were analyzed by hematoxylin/eosin (H&E), and Sirius Red staining, respectively. The atherosclerotic plaque, or Sirius Red-stained area (refer to collagen content) in a given image was highlighted and quantified ($pixel^2$ for lesion size; percentage over the atherosclerotic lesion area for collagen content) by two experienced investigators blinded to the treatments using Image J pro software. Three to six sections were analyzed per aortic roots (or per mouse) and averaged.

Immunofluorescence analysis of human femoral and coronary arteries

For immunofluorescence analysis, paraffin sections of human femoral and coronary arteries were deparaffined with xylene and rehydrated with ethanol, and then incubated with Tris-EDTA Buffer at 95~100˚C for 30 minutes to retrieve antigens. After blocking with 10% normal matched serum (Dako), the sections were incubated with mouse anti-SMαA antibody (1:500 dilutions) and rabbit anti-Cezanne antibody (1:100), or mouse/rabbit IgG control diluted in blocking buffer at 4°C overnight. After washed, sections were incubated with an appropriate Alexa Fluor Plus 488/594/647-conjugated 2nd antibody (ThermoFisher UK, 1:1000 dilution), followed by nuclei staining with 4,6-diamidino-2-phenylindole (DAPI) (1ug/ml). After mounting, the slides were examined using a laser scanning confocal microscope (Zeiss LSM 510 Mark 4) and Zen 2009 image software. The mean fluorescence intensity (MFI) for red (Cezanne) and blue (DAPI) fluorescence signal of the human femoral arterial wall, and the plaque shoulder (PS) or adjacent regions (AR) of human coronary atherosclerotic plaques) from each image was measured with Image J pro software by two experienced investigators blinded to the treatments, and presented as the relative MFI (target proteins over DAPI).

Supplementary table S1: Primer sets used in the present study

Gene names	Forward (5’-3’)	Reverse (5’-3’)	Application
18s (mus/hu)	AAACGGCTACCACATCCAAG	CCTCCAATGGATCCTCGTTA	RT-qPCR
Cezanne/Otud7b (mus)	TGTCCGATTGGCCAGTATAA	ACAGTGGGATCCACTTCACA	RT-qPCR
Cezanne/Otud7b (hu)	ACAATGTCCGATTGGCCAGT	ACAGTGGGATCCACTTCACATTC	RT-qPCR
Cezanne/Otud7b (mus/hu)	TGGGAGTGGGGCAAAGATGA	ACAGTGGGATCCACTTCACA	RT-qPCR
Mus SMαA	TCCTGACGCTGAAGTATCCGAT	GGCCACACGAAGCTCGTTATAG	RT-qPCR
Mus h1-Calponin	GGT CCT GCC TAC GGC TTG TC	TCG CAA AGA ATG ATC CCG TC	RT-qPCR
Mus SM-myh11	AAG CAG CCA GCA TCA AGG AG	AGC TCT GCC ATG TCC TCC AC	RT-qPCR
Mus SM22α	GATATGGCAGCAGTGCAGAG	AGTTGGCTGTCTGTGAAGTC	RT-qPCR
Mus SMTN-B	GGGCAGTATCTTCGACCGAG	GGCAGACTCTGTGCCTTCAT	RT-qPCR
Mus CCN1/Cyr61	AGAGGCTTCCTGTCTTTGGC	CCAAGACGTGGTCTGAACGA	RT-qPCR
Mus CCN2/Ctgf	AGAACTGTGTACGGAGCGTG	GTGCACCATCTTTGGCAGTG	RT-qPCR
Mus CCN5/Wisp2	GCCCAAGGACACCAACTTTCT	GGTTCTGGTTGGATACTCGGG	RT-qPCR
Mus Ctnnb1	CGGGCCTGAGGGTACCTG	GGCCATGTCCAACTCCATCA	RT-qPCR
Mus PCNA	TTGCACGTATATGCCGAGACCT	ATTGCCAAGCTCTCCACTTGC	RT-qPCR
Mus Ki67	AGAGCTAACTTGCGCTGACT	GGAGAAGCCTCTCGGTGAAG	RT-qPCR
Mus MCP-1	CCCCAAGAAGGAATGGGTCC	TGCTTGAGGTGGTTGTGGAA	RT-qPCR
Mus iNOS/NOS2	GCCACCAACAATGGCAACAT	TCGATGCACAACTGGGTGAA	RT-qPCR
Mus ICAM-1	GTGGGTCGAAGGTGGTTCTT	AAACAGGAACTTTCCCGCCA	RT-qPCR
Mus VCAM-1	TTCTGACGTGTGCTGCTATTGG	TTTGGCCCCCTCATTCCTT	RT-qPCR
Mus E-selectin	AACCTCACTCCTGACATCGTCC	ATTGAAGGCTTTGGCAGCTG	RT-qPCR
pGL3-CCN1-WRE^{mut}	TCCATGTGTTTAACT*CATGTACC*TTGGATCACTTAG	CTAAGTGATCCAA*GGTACATG*AGTTAAACACATGGA	WRE mutation in murine CCN1 hene promoter
Mus CCN1 gene promoter	CCTATGTGTCTCGCTGTGTG	AGCCACTGCACTACCCTTG	CHIP
Mus CCN1 gene intron	AAGCGCGTGAGATTGCTTTG	GAGACACTTCTGGTGGACGG	CHIP

Reference:

1. Chen Q, Yang F, Guo M, Wen G, Zhang C, Luong le A, Zhu J, Xiao Q and Zhang L. miRNA-34a reduces neointima formation through inhibiting smooth muscle cell proliferation and migration. *Journal of molecular and cellular cardiology*. 2015;89:75-86.
2. Afzal TA, Luong LA, Chen D, Zhang C, Yang F, Chen Q, An W, Wilkes E, Yashiro K, Cutillas PR, Zhang L and Xiao Q. NCK Associated Protein 1 Modulated by miRNA-214 Determines Vascular Smooth Muscle Cell Migration, Proliferation, and Neointima Hyperplasia. *Journal of the American Heart Association*. 2016;5.
3. Zhang L, Chen Q, An W, Yang F, Maguire EM, Chen D, Zhang C, Wen G, Yang M, Dai B, Luong LA, Zhu J, Xu Q and Xiao Q. Novel Pathological Role of hnRNPA1 (Heterogeneous Nuclear Ribonucleoprotein A1) in Vascular Smooth Muscle Cell Function and Neointima Hyperplasia. *Arteriosclerosis, thrombosis, and vascular biology*. 2017;37:2182-2194.
4. Yang F, Chen Q, He S, Yang M, Maguire EM, An W, Afzal TA, Luong LA, Zhang L and Xiao Q. miR-22 Is a Novel Mediator of Vascular Smooth Muscle Cell Phenotypic Modulation and Neointima Formation. *Circulation*. 2018;137:1824-1841.
5. Xiao Q, Zhang F, Grassia G, Hu Y, Zhang Z, Xing Q, Yin X, Maddaluno M, Drung B, Schmidt B, Maffia P, Ialenti A, Mayr M, Xu Q and Ye S. Matrix metalloproteinase-8 promotes vascular smooth muscle cell proliferation and neointima formation. *Arteriosclerosis, thrombosis, and vascular biology*. 2014;34:90-8.
6. Zhang C, Chen D, Maguire EM, He S, Chen J, An W, Yang M, Afzal TA, Luong LA, Zhang L, Lei H, Wu Q and Xiao Q. Cbx3 inhibits vascular smooth muscle cell proliferation, migration, and neointima formation. *Cardiovascular research*. 2018;114:443-455.
7. Evans PC, Smith TS, Lai MJ, Williams MG, Burke DF, Heyninck K, Kreike MM, Beyaert R, Blundell TL and Kilshaw PJ. A novel type of deubiquitinating enzyme. *J Biol Chem*. 2003;278:23180-6.
8. Zhao H, Wen G, Huang Y, Yu X, Chen Q, Afzal TA, Luong LA, Zhu J, Shu Y, Zhang L and Xiao Q. MicroRNA-22 Regulates Smooth Muscle Cell Differentiation From Stem Cells by Targeting Methyl CpG-Binding Protein 2. *Arteriosclerosis, thrombosis, and vascular biology*. 2015.
9. Luo Z, Wen G, Wang G, Pu X, Ye S, Xu Q, Wang W and Xiao Q. MicroRNA-200C and -150 play an important role in endothelial cell differentiation and vasculogenesis by targeting transcription repressor ZEB1. *Stem Cells*. 2013;31:1749-62.
10. Yu X, Zhang L, Wen G, Zhao H, Luong LA, Chen Q, Huang Y, Zhu J, Ye S, Xu Q, Wang W and Xiao Q. Upregulated sirtuin 1 by miRNA-34a is required for smooth muscle cell differentiation from pluripotent stem cells. *Cell Death Differ*. 2014.
11. Huang Y, Lin L, Yu X, Wen G, Pu X, Zhao H, Fang C, Zhu J, Ye S, Zhang L and Xiao Q. Functional involvements of heterogeneous nuclear ribonucleoprotein A1 in smooth muscle differentiation from stem cells in vitro and in vivo. *Stem Cells*. 2013;31:906-17.
12. Xiao Q, Zhang F, Lin L, Fang C, Wen G, Tsai TN, Pu X, Sims D, Zhang Z, Yin X, Thomaszewski B, Schmidt B, Mayr M, Suzuki K, Xu Q and Ye S. Functional role of matrix metalloproteinase-8 in stem/progenitor cell migration and their recruitment into atherosclerotic lesions. *Circulation research*. 2013;112:35-47.

13. Fang C, Wen G, Zhang L, Lin L, Moore A, Wu S, Ye S and Xiao Q. An important role of matrix metalloproteinase-8 in angiogenesis in vitro and in vivo. *Cardiovascular research*. 2013;99:146-55.

14. Luong le A, Fragiadaki M, Smith J, Boyle J, Lutz J, Dean JL, Harten S, Ashcroft M, Walmsley SR, Haskard DO, Maxwell PH, Walczak H, Pusey C and Evans PC. Cezanne regulates inflammatory responses to hypoxia in endothelial cells by targeting TRAF6 for deubiquitination. *Circulation research*. 2013;112:1583-91.

15. Wen G, An W, Chen J, Maguire EM, Chen Q, Yang F, Pearce SWA, Kyriakides M, Zhang L, Ye S, Nourshargh S and Xiao Q. Genetic and Pharmacologic Inhibition of the Neutrophil Elastase Inhibits Experimental Atherosclerosis. *Journal of the American Heart Association*. 2018;7.

Supplementary Figures and Figure Legend:

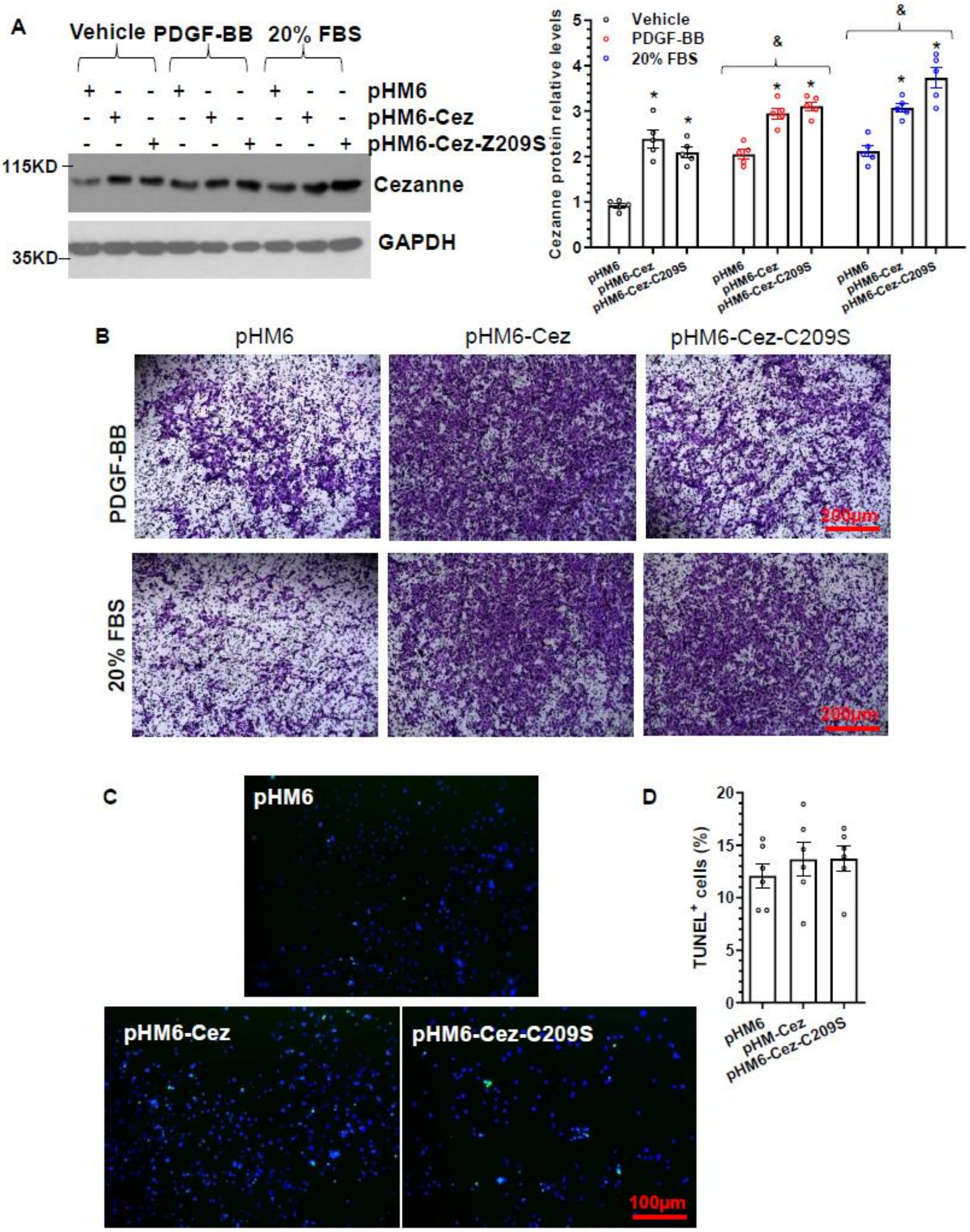

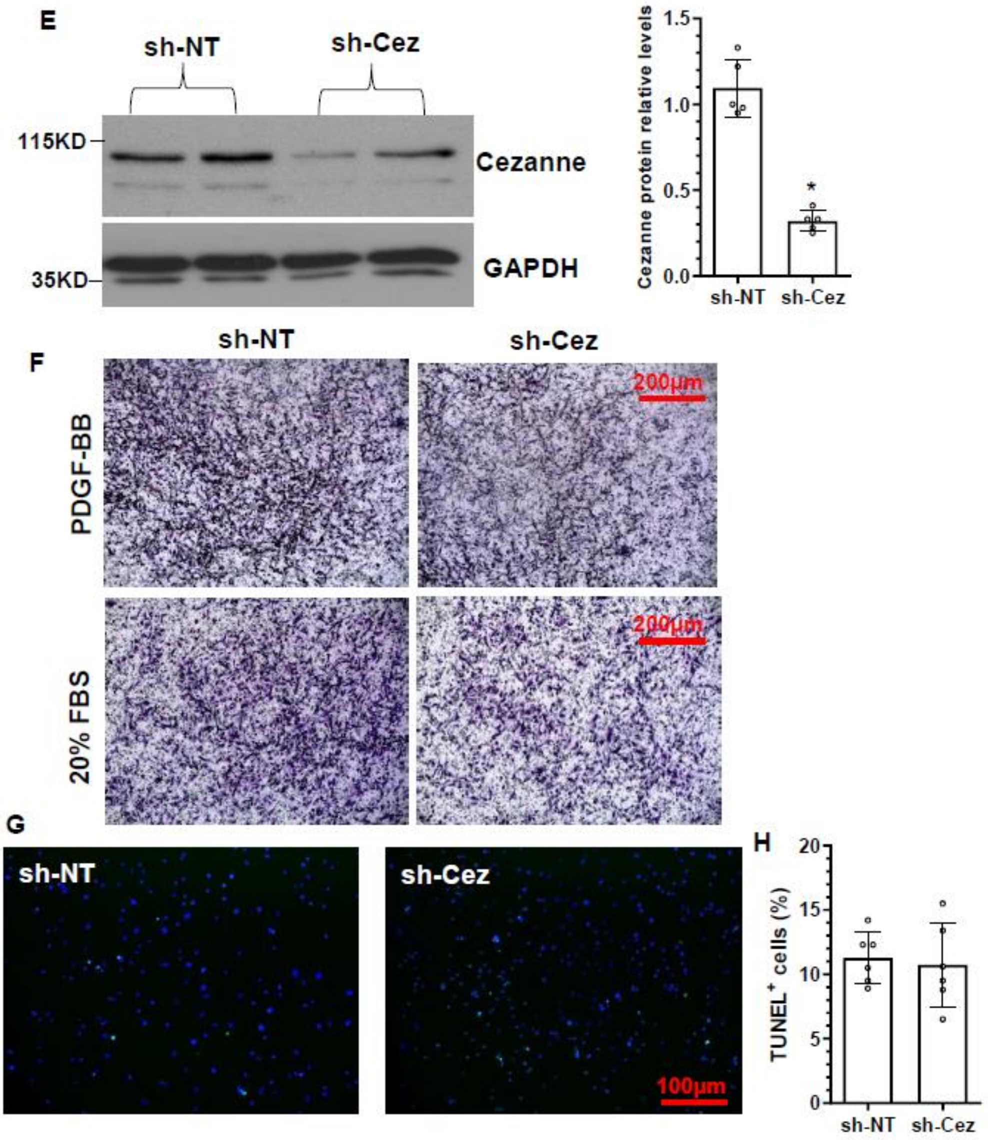

Figure S1. Cezanne mediates VSMC functions. (A & E) Western blot analysis of Cezanne protein. (B & F) Representative images for transwell migration assay described in Figure 2D & 2H. (C-D & G-H) TUNEL analysis of VSMC apoptosis. VSMCs transfected with respective plasmids or infected with shRNA lentivirus as indicated were subjected to serum starvation for 48 hours, followed by 100ng/ml TNFα and 200ng/ml IFNγ stimulations for another 72 hours to induce apoptosis, which was evaluated using TUNEL analysis. The data presented here are representative (Left panel in A & E, B-C, F-G) or mean±S.E.M. (Right panel in A & E, D & H) of five (n=5, A & E) or six (n=6, B-D & F-H) independent experiments. *P<0.05 (versus pHM6 or sh-NT); $^{\&}$P<0.05 (versus vehicle) (E, unpaired *t*-test; A, two-way ANOVA with a *post hoc* test of Tukey's analysis).

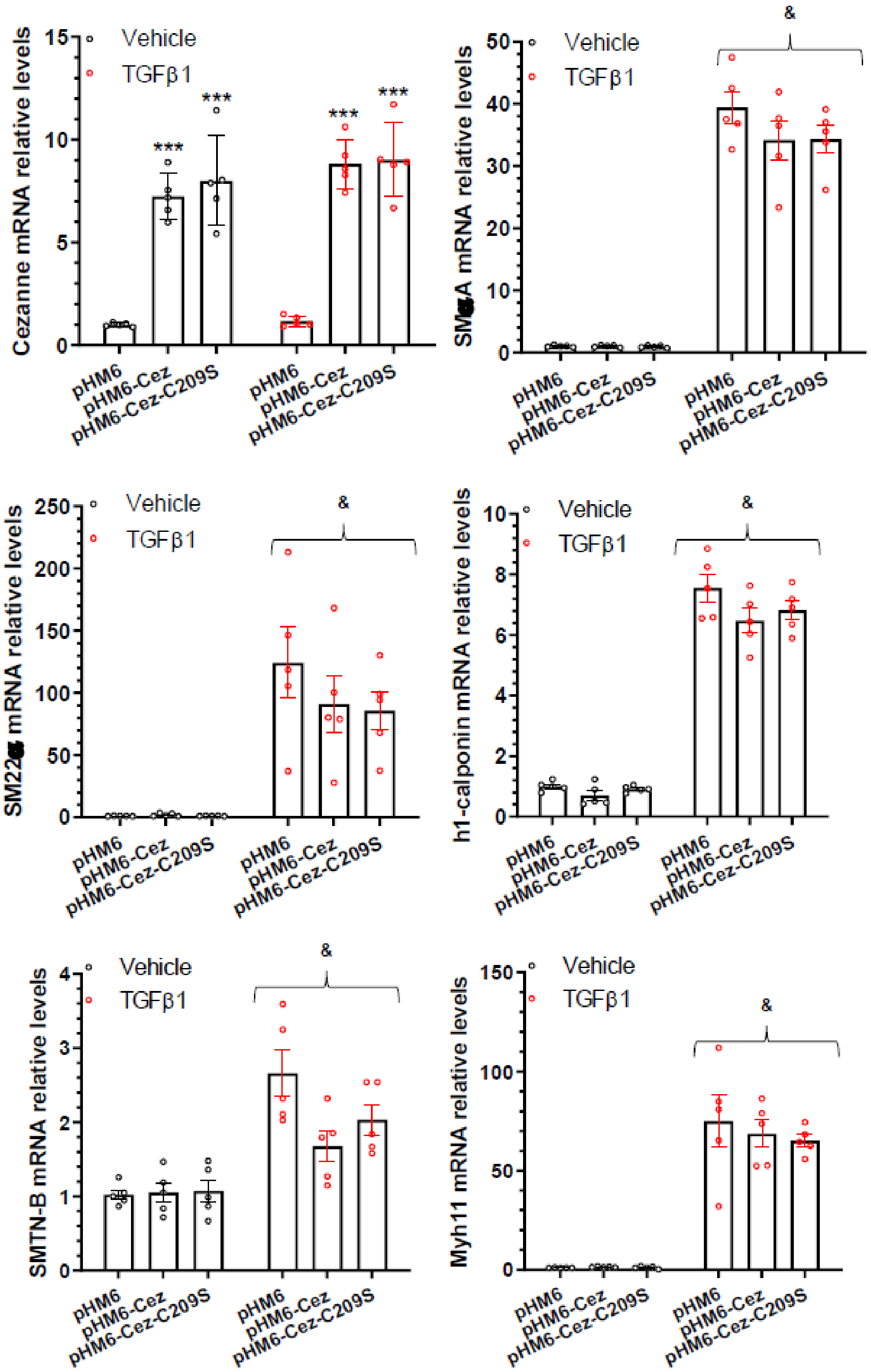

Figure S2. VSMC genes expression were not affected by Cezanne. VSMCs transfected with control (pHM6), wild-type (pHM6-Cez) or mutated (pHM6-Cez-C209S) Cezanne plasmids were subjected to serum starvation for 48 hours, followed by 5ng/ml TGFβ1 stimulation for additional 12 hours. The data presented here are mean±S.E.M. of five independent experiments (n=5). ***$P<0.001$ (versus pHM6); $^{\&}P<0.05$ (versus vehicle) (two-way ANOVA with a *post hoc* test of Tukey's analysis).

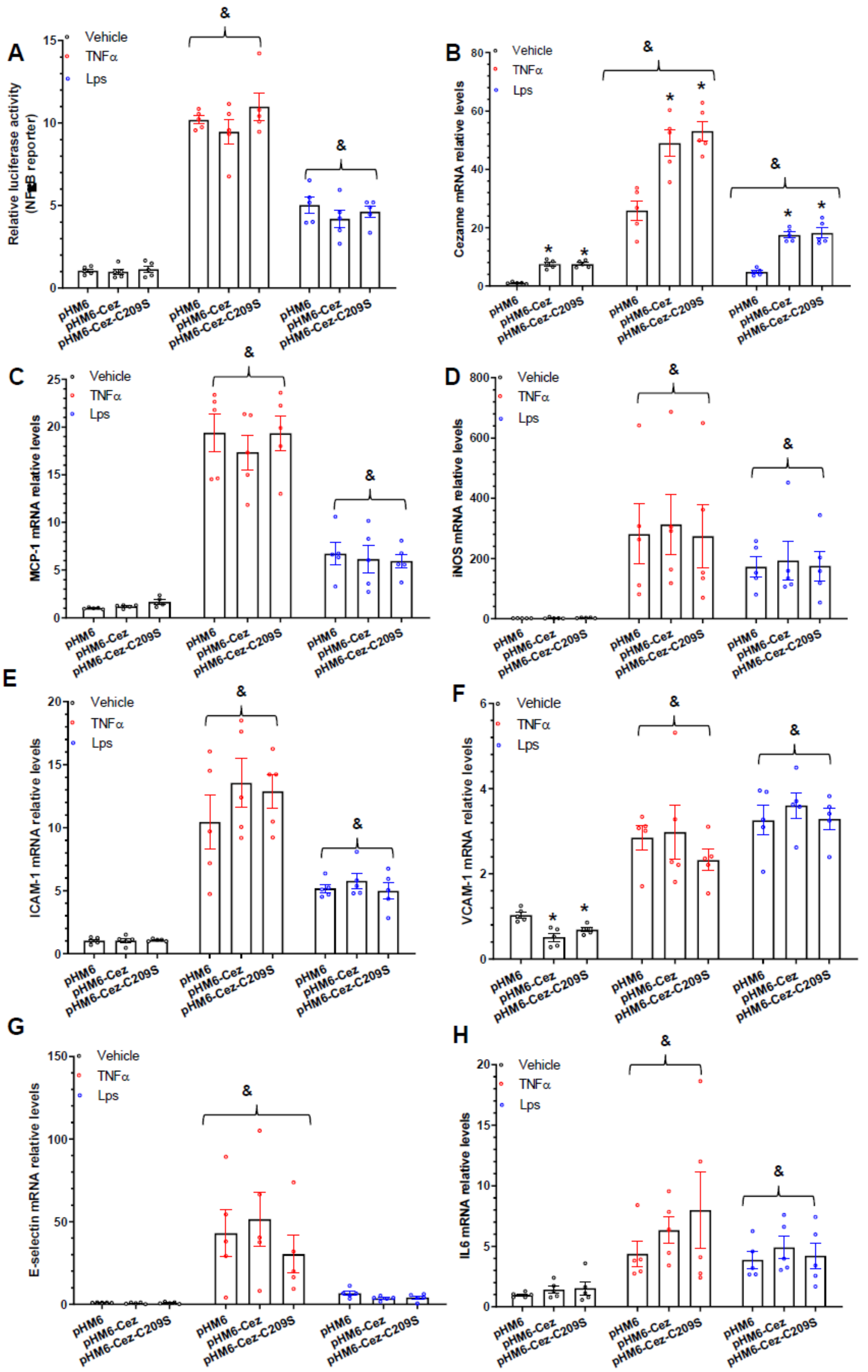

Figure S3. Cezanne plays nonsignificant role in NF-kB signalling and inflammatory response in VSMCs. (A) Luciferase activity assays with NF-kB reporter. VSMCs co-transfected with NF-kB reporter and control (pHM6), wild-type (pHM6-Cez) or mutated (pHM6-Cez-C209S) Cezanne plasmids were subjected to serum starvation for 48 hours, followed by 50ng/ml TNFα or 1000ng/ml Lps stimulation for additional 6 hours. Cell lysates were harvested and subjected to luciferase activity assay. (B-H) RT-qPCR analysis. VSMCs

transfected with control (pHM6), wild-type (pHM6-Cez) or mutated (pHM6-Cez-C209S) Cezanne plasmids were subjected to serum starvation for 48 hours, followed by 50ng/ml TNFα or 1000ng/ml Lps stimulation for additional 6 hours. Total RNAs were harvested and subjected to RT-qPCR analysis with the indicated gene-specific primers. The data presented here are mean±S.E.M. of five independent experiments (n=5). *P<0.05 (versus pHM6); $^{\&}$P<0.05 (versus vehicle) (two-way ANOVA with a *post hoc* test of Tukey's analysis).

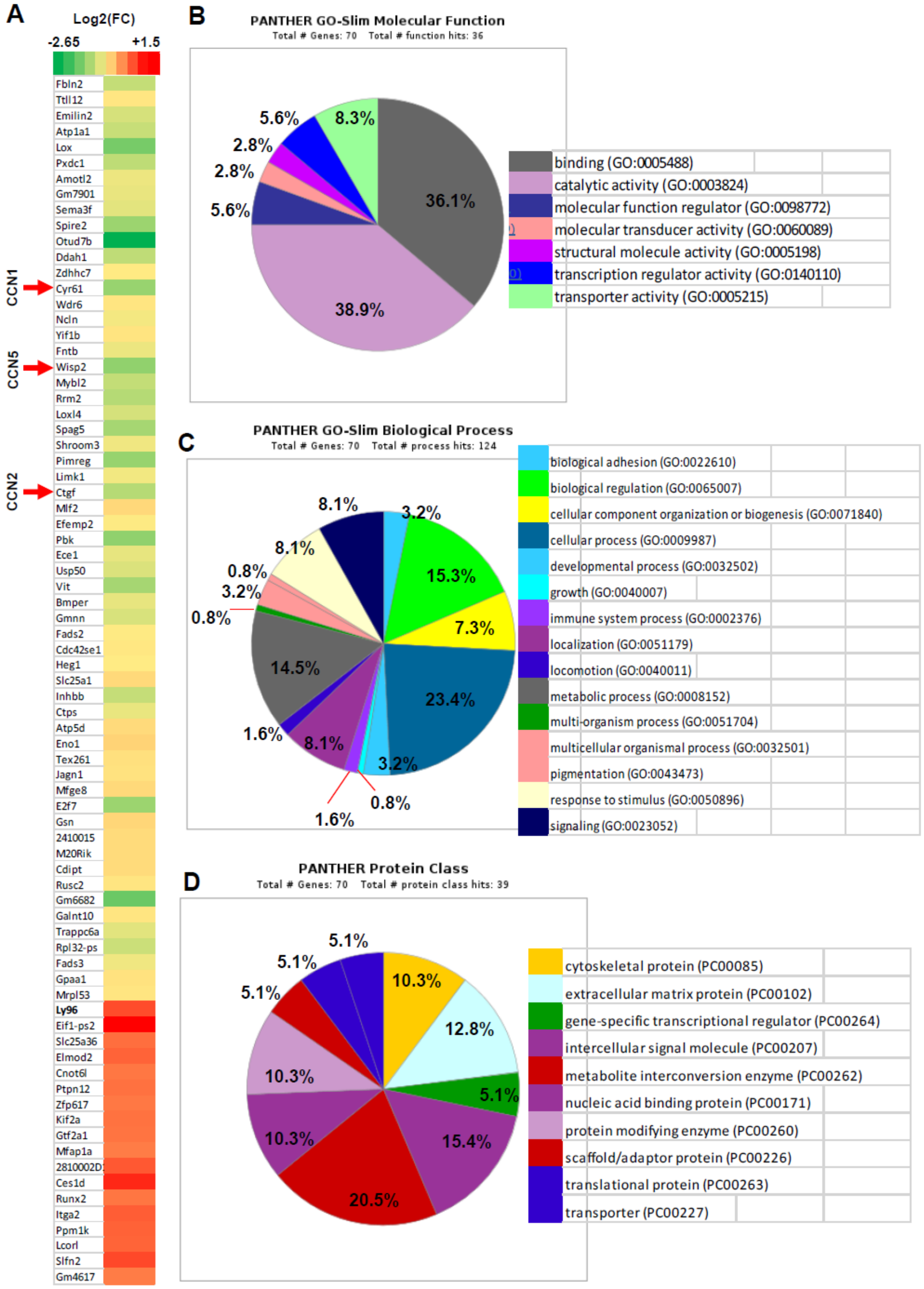

E **PANTHER Overrepresentation Test with the Bonferroni correction for multiple testing**

PANTHER GO-Slim Molecular Function	Fold Enrichment	+/-	Corrected P value
carbohydrate derivative binding	5.26	+	0.00746
heparin binding	35.34	+	0.000113
integrin binding	34.08	+	0.000124
cell adhesion molecule binding	13.63	+	0.00157
protein-containing complex binding	4.5	+	0.0126
PANTHER GO-Slim Biological Process	Fold Enrichn	+/-	P value
cellular protein modification process	2.22	+	0.0277
supramolecular fiber organization	5.39	+	0.00685
actin cytoskeleton organization	4.01	+	0.04
regulation of cell migration	8.05	+	0.0268
cell death	5.13	+	0.0216
cell adhesion	4.42	+	0.0134
PANTHER Protein Class	Fold Enrichn	+/-	P value
Runt transcription factor	> 100	+	0.0125
growth factor	14.14	+	0.000218
intercellular signal molecule	5.56	+	0.000788
extracellular matrix structural protein	13.07	+	0.00176
extracellular matrix protein	9.88	+	0.000175
general transcription factor	6.56	+	0.0387
actin or actin-binding cytoskeletal protein	4.04	+	0.0392
Unclassified	0.8	-	0

Figure S4. Genes and signal pathways were regulated by Cezanne in VSMCs. (**A**) Heatmap showing 75 genes were significantly regulated by Cezanne gene knockdown in VSMCs as determined by RNA sequencing analysis. (B-D) Panther analysis of the genes shown in the Heatmap (A) based on GO-slim molecular function (B), GO-Slim biological process (C), or protein class (D), respectively. (E) Panther overrepresentation analysis of the genes shown in the Heatmap (A).

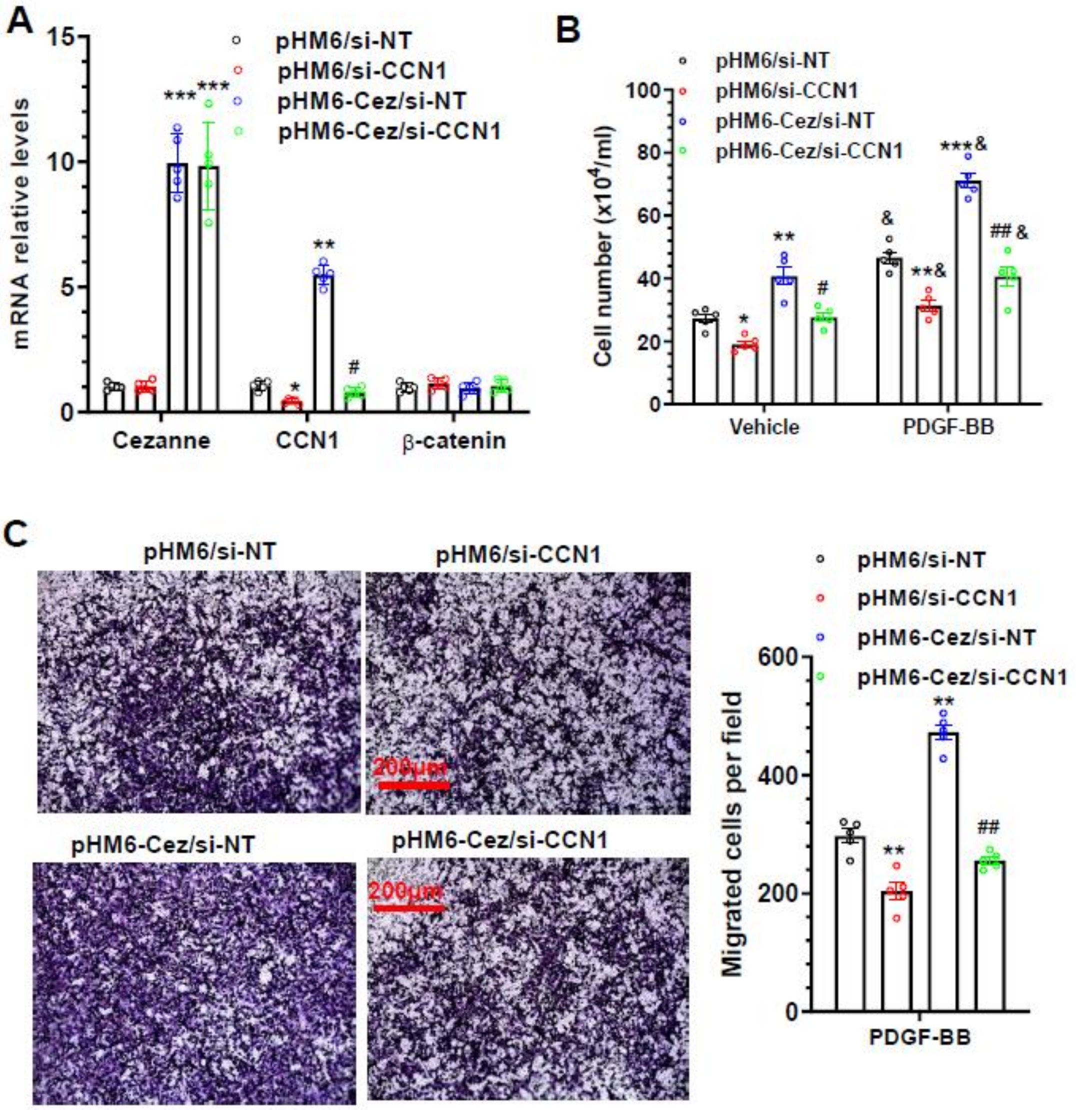

Figure S5. Silencing CCN1 gene expression abolished the promotive effects of Cezanne over-expression on VSMC proliferation and migration. VSMCs co-transfected with control (pHM6) or Cezanne (pHM6-Cez) plasmids and control (si-NT) or CCN1 specific (si-CCN1) siRNAs were subjected to serum starvation for 48 hours, followed by 10ng/ml PDGF-BB stimulations for another 12 (migration) or 48 (cell counting) hours, respectively. RT-qPCR (A), cell counting (B), and transwell migration (C) assays were conducted to examine gene expression and respective cellular functions. The data presented here are representative (left, C) or mean±S.E.M. of five independent experiments (n=5). *P<0.05, **<0.01, ***<0.001 (versus pHM6/si-NT); #P<0.05, # #<0.01 (versus pHM6-Cez/si-NT); &P<0.05 (versus vehicle) (one- (A & C) or two-way (B) ANOVA with a *post hoc* test of Tukey's analysis).

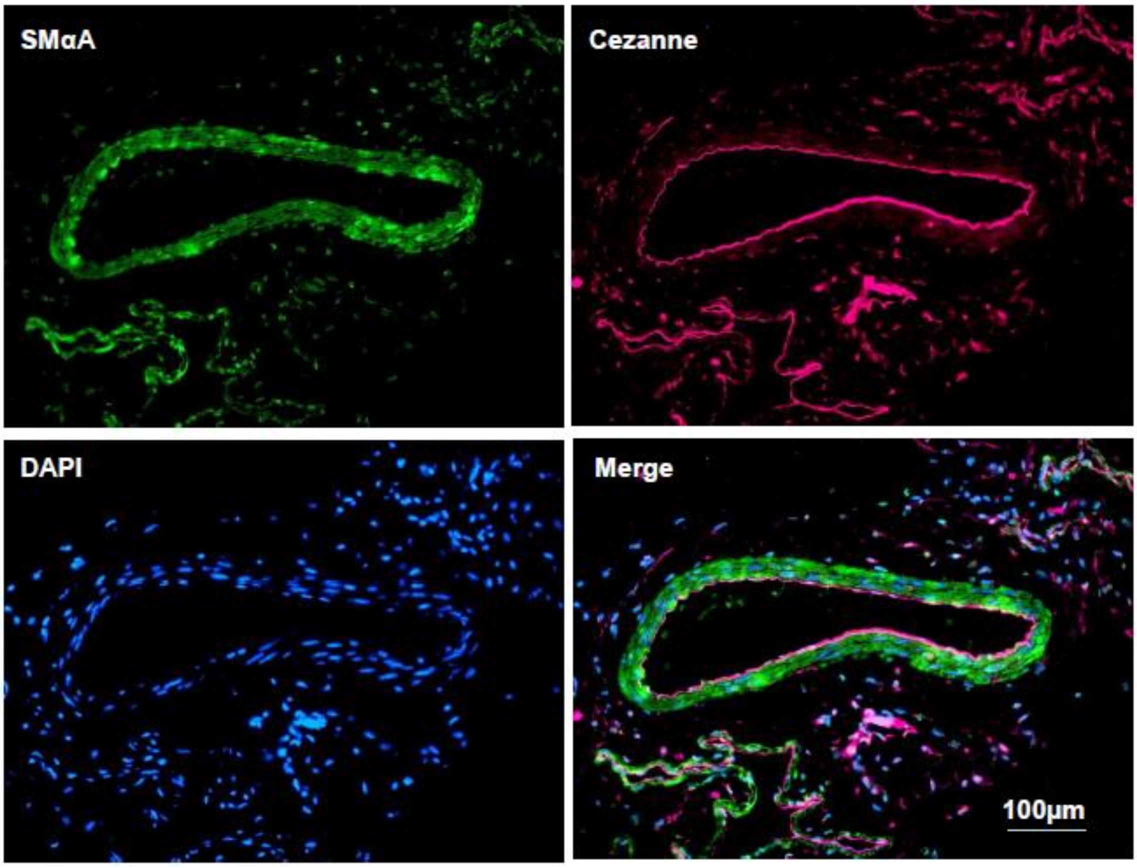

Figure S6. Cezanne expression in normal/uninjured murine aorta. Immunostaining analysis showing Cezanne is highly expressed in endothelium in normal murine aorta. Representative images from three mice were presented here.

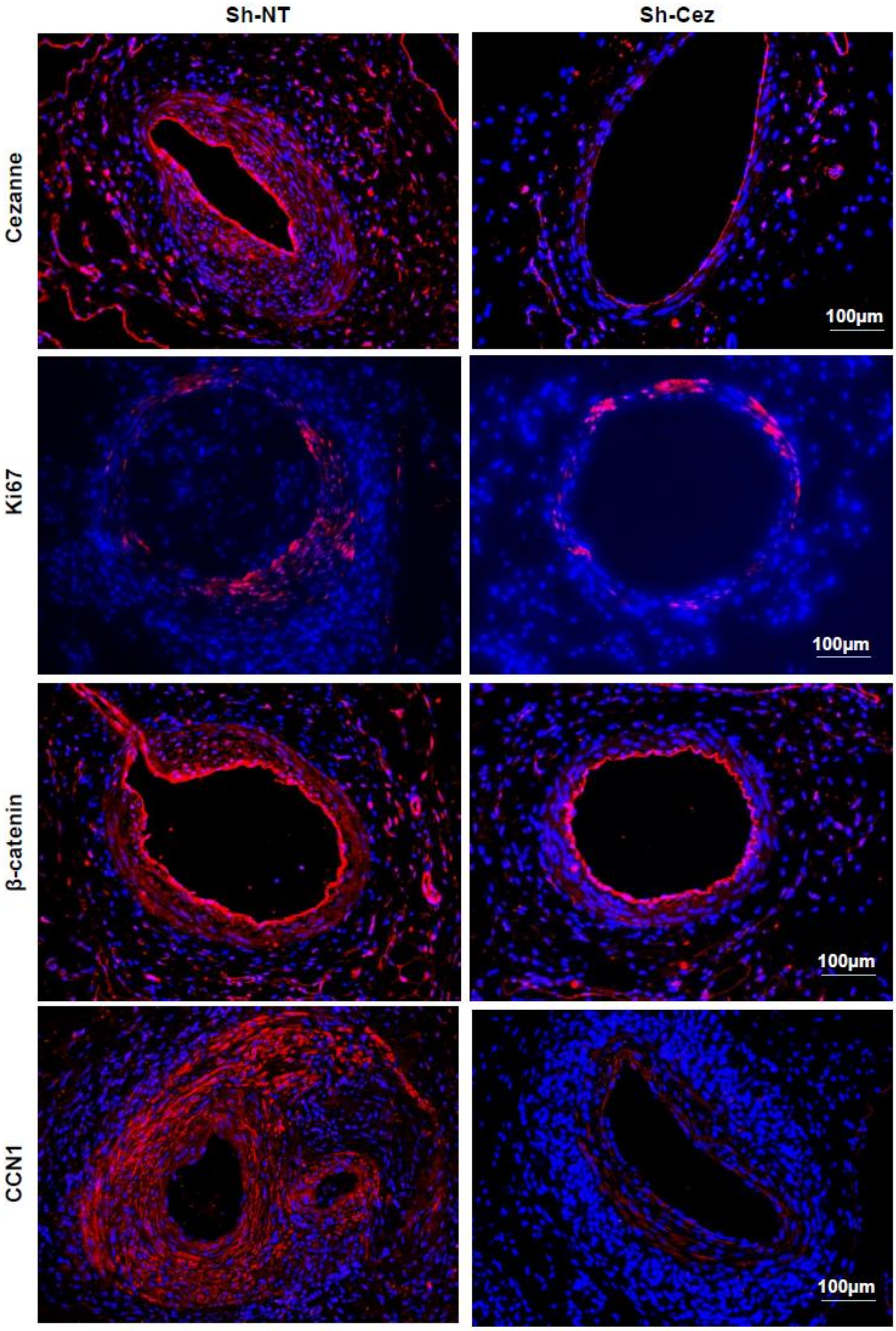

Figure S7. Immunostaining analysis. After injury, 10~20μl of DMEM containing 1.0~2.0 x 10^6 shRNA lentiviral particles (sh-NT or sh-Cez) was directly infused into the lumen of the injured femoral arteries, followed by a 30-minute incubation for local VSMC infection. At 14 days post-treatment, injured arterial segments were harvested and subjected to immunofluorescence staining analyses with the indicated antibodies. Representative images from six mice were presented here.

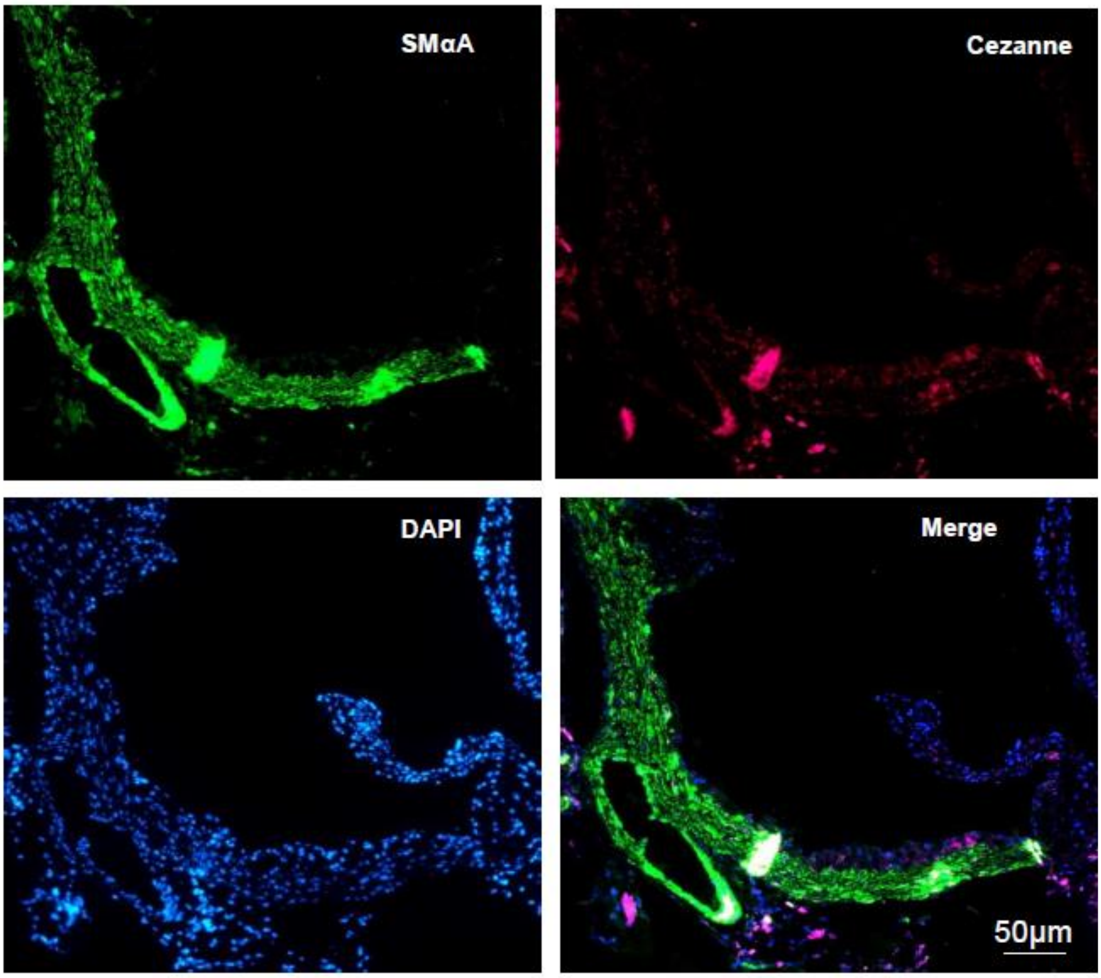

Figure S8. Cezanne expression in normal murine aortic roots. Aortic roots without atherosclerotic plaques were harvested from eight week old male $Cez^{+/+}/ApoE^{-/-}$ mice and subjected to immunostaining analysis. Representative images from three mice were presented here.

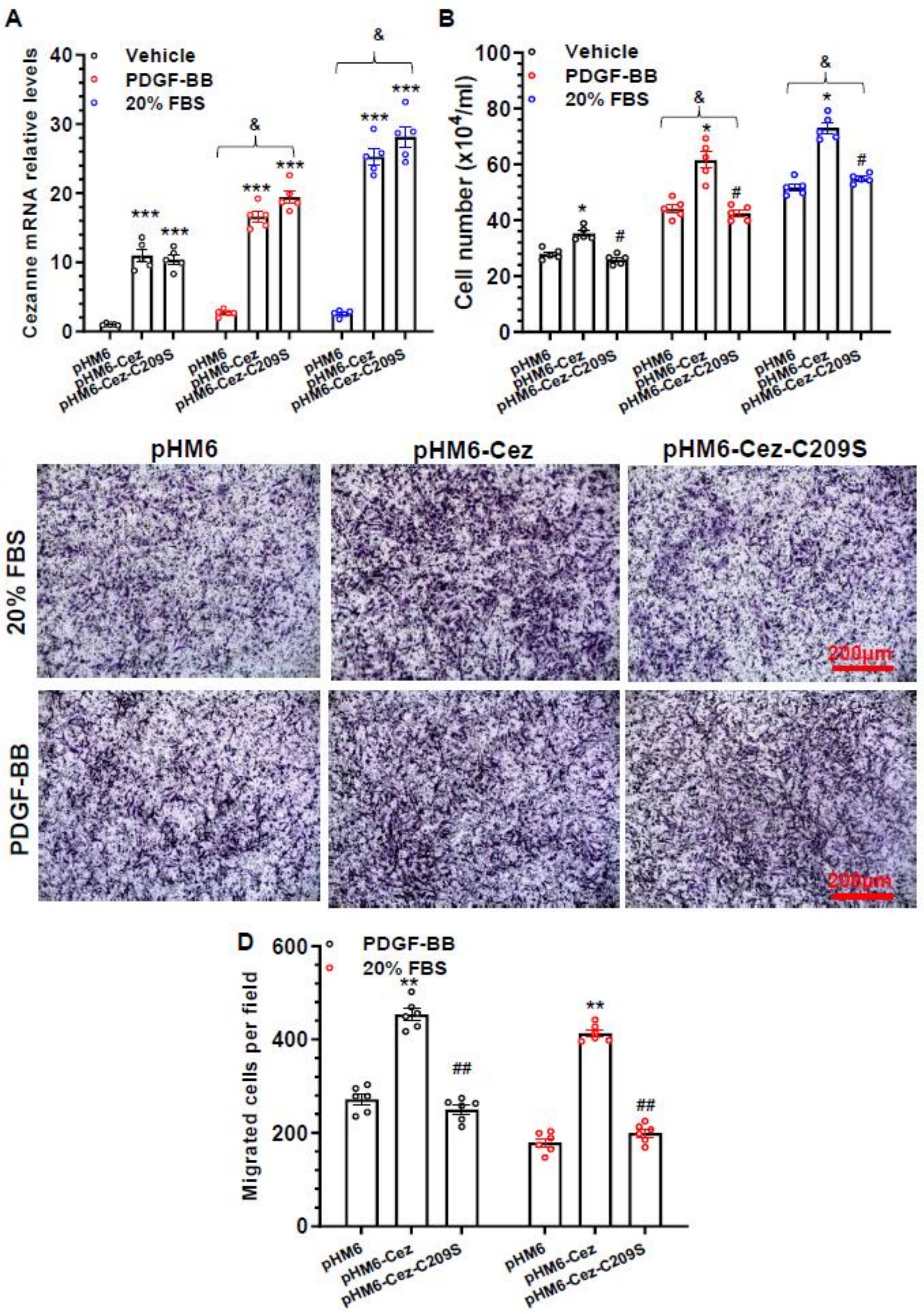

Figure S9. Cezanne regulates human VSMC functions. Human aorta SMCs transfected with control (pHM6), wild-type (pHM6-Cez) or mutated (pHM6-Cez-C209S) Cezanne plasmids were subjected to serum starvation for 48 hours, followed by 10ng/ml PDGF-BB, or 20%FBS stimulations for another 12 (migration) or 48 (cell counting) hours, respectively. RT-qPCR (A), cell counting (B), and transwell migration (C & D) assays were conducted to examine gene expression and respective cellular functions. The data presented here are representative (C) or mean±S.E.M. of five (n=5 in A-B) or six (n=6 in C-D) independent experiments. *P<0.05, **<0.01, ***<0.001 (versus pHM6); $^{\#}$P<0.05, $^{\#\#}$<0.01 (versus pHM6-Cez); $^{\&}$P<0.05 (versus vehicle) (two-way ANOVA with a *post hoc* test of Tukey's analysis).

Unedited gel for each representative western blot shown in the manuscript:

Figure 1B_Cezanne

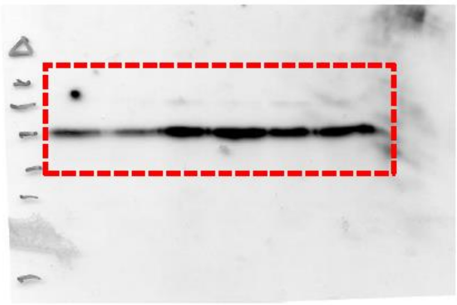

Figure 1B_α-tubulin

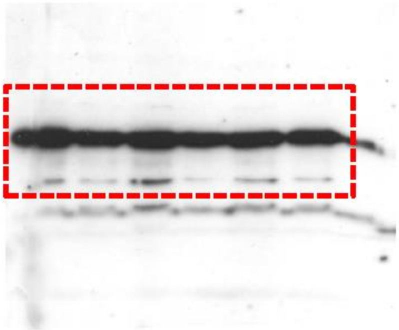

Figure 3D_IP-Ub & WB-CCN1

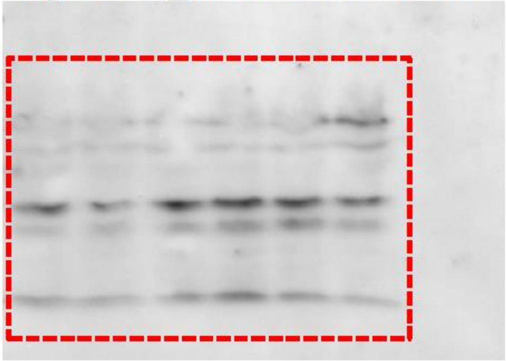

Figure 3D_WB-CCN1 (cell lysate)

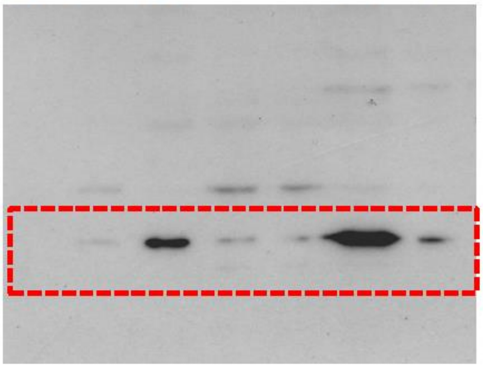

Figure 3D_WB-GAPDH (cell lysate)

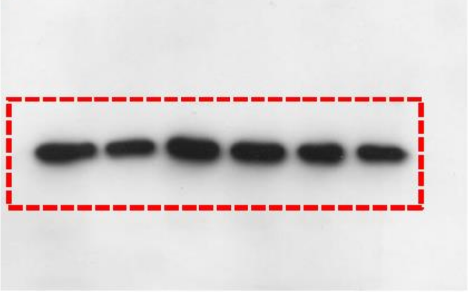

Figure 4B_β-catenin (WB) & Ub (IP)

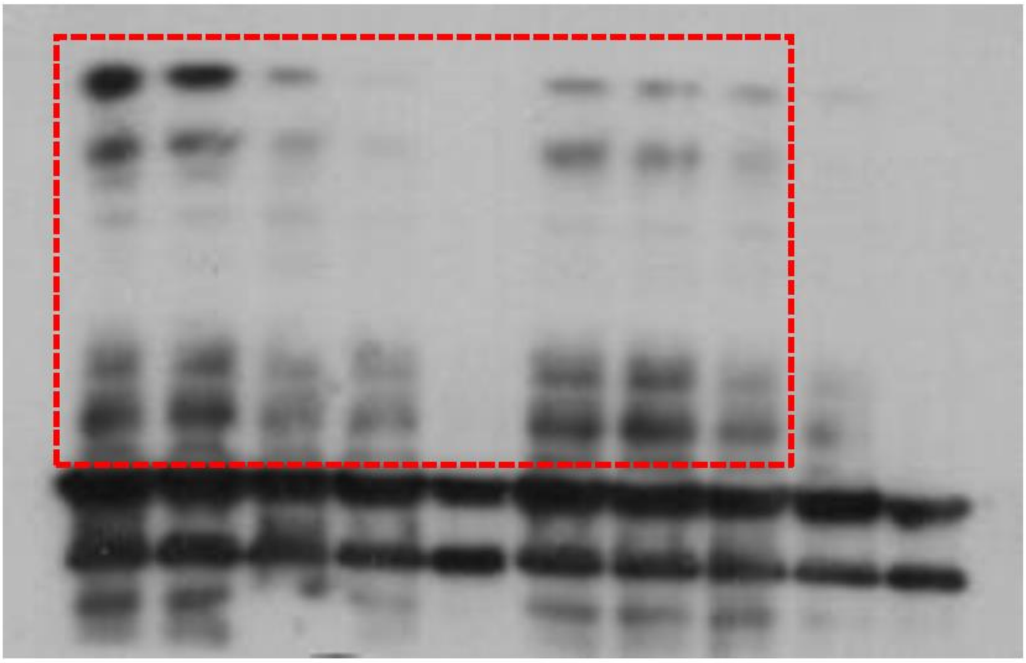

Figure 4B_β-catenin (WB, cell lysate)

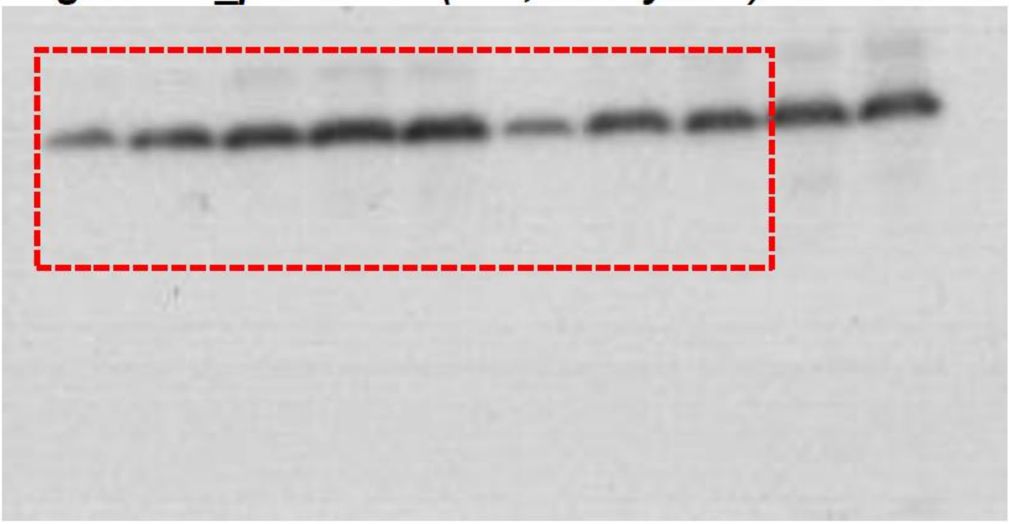

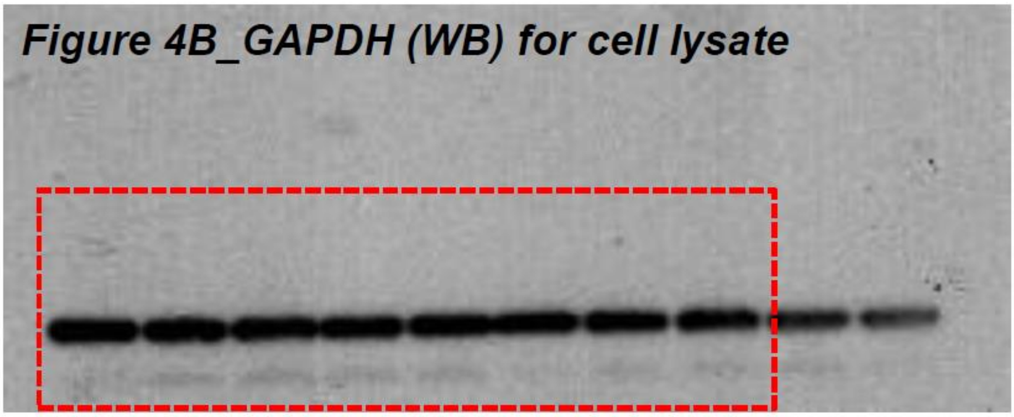

Figure 4B_GAPDH (WB) for cell lysate

Figure 5B_Cezanne

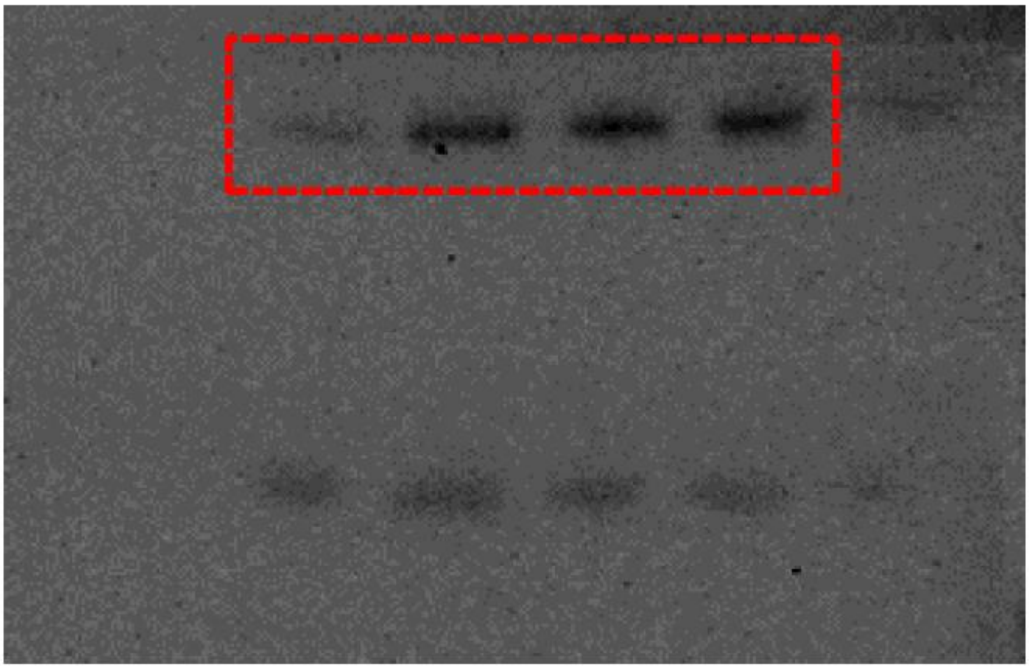

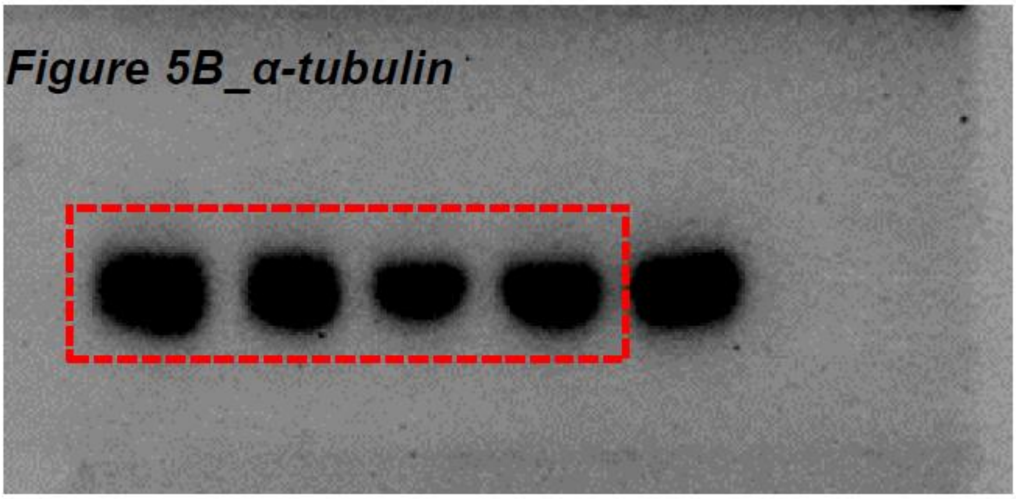

Figure S1A_Cezanne

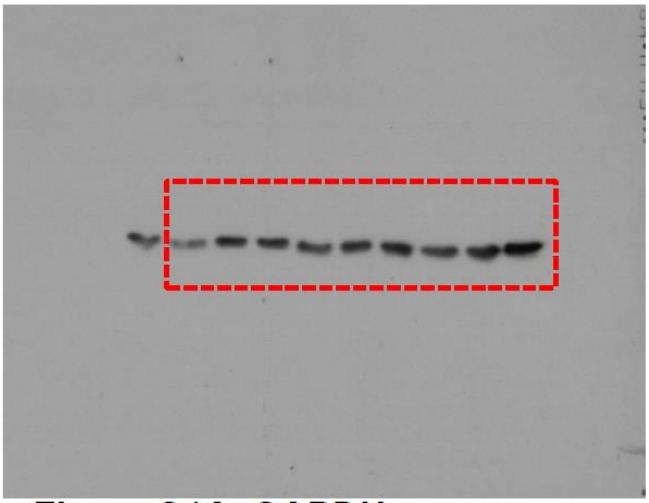

Figure S1A_GAPDH

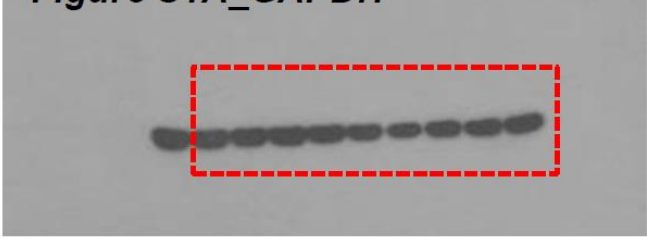

Figure S1E_Cezanne

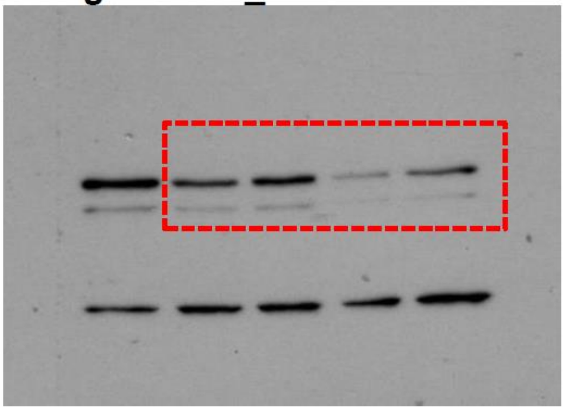

Figure S1E_GAPDH

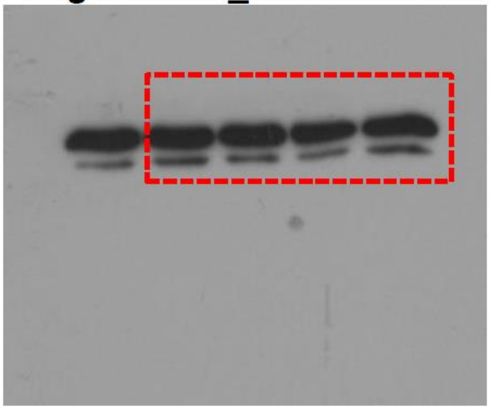

Supplement: cvab056_Supplementary_Data [file cvab056_supplementary_data.pdf]
